# Modulation of Physiological Responses in Damask Rose to Combined Salinity and *Botrytis cinerea* Stress by Chitosan and Nano‐Chelated Copper

**DOI:** 10.1002/pei3.70243

**Published:** 2026-11-10

**Authors:** Mina Khorrami Moghaddam, Nima Akbari Oghaz

**Affiliations:** ^1^ Department of Horticultural Sciences, Faculty of Plant Production Gorgan University of Agricultural Sciences and Natural Resources Gorgan Golestan Iran; ^2^ Department of Plant Protection, Faculty of Plant Production Gorgan University of Agricultural Sciences and Natural Resources Gorgan Golestan Iran

**Keywords:** antioxidant enzymes, calcium chloride, leaf pigments, sodium chloride

## Abstract

Salinity exacerbates *Botrytis cinerea* damage, posing a major constraint to *Rosa × damascena* greenhouse cultivation. This study investigated the physio‐biochemical responses of *R. × damascena* to individual and combined salinity (50 and 100 mM NaCl/CaCl_2_) and *B. cinerea* stress, evaluating the mitigating potential of foliar‐applied chitosan (0.4% w/v) and nano‐chelated copper (NCC; 1000 and 2000 mg L^−1^). Using a four‐way factorial design (36 treatments; *N* = 108), we assessed morphological traits, photosynthetic pigments, proline content, antioxidant enzyme activities (superoxide dismutase, peroxidase, catalase), and membrane integrity via electrolyte leakage (EC). Combined stress resulted in nonadditive physiological deterioration, characterized by significantly elevated EC and disease indices alongside suppressed antioxidant capacity and pigment retention compared to individual stressors. While salinity alone induced dose‐dependent osmotic adjustments, its combination with *B. cinerea* overwhelmed host defense mechanisms, leading to severe membrane destabilization. Both chitosan and NCC applications partially restored physiological homeostasis by enhancing superoxide dismutase and peroxidase activities, reducing electrolyte leakage, and preserving chlorophyll and carotenoid levels. The co‐application of chitosan and NCC demonstrated superior efficacy, particularly under moderate combined stress, by synergistically improving membrane stability and reducing leaf and flower disease severity. However, antioxidant responses were context‐dependent, with catalase activity remaining largely unaffected, suggesting a partial rather than complete restoration of redox balance. These findings indicate that while chitosan and NCC offer promising mitigation strategies against compounded abiotic–biotic stress, their efficacy is limited by stress intensity. Future work must validate environmental safety, ion dynamics, and field efficacy to ensure sustainable rose production.

## Graphical abstract

Co‐application of nano‐chelated copper and chitosan mitigates salinity and gray mold stress in *Rosa* × *damascena* by enhancing antioxidant enzymatic activity, preserving membrane integrity, partially restoring chlorophyll content, and consistently reducing leaf and flower disease severity; though physiological recovery remains dependent on stress intensity and treatment concentration.

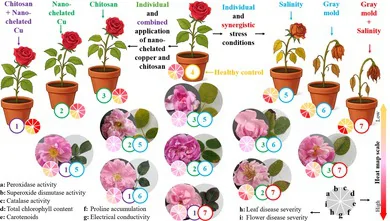

## 1 Introduction

*Rosa × damascena* Mill., a globally significant ornamental and medicinal species (Wang 2024), faces escalating production challenges due to intensified cultivation practices that heighten susceptibility to phytopathogens, particularly the necrotrophic fungus *Botrytis cinerea* Pers. (1794). This pathogen causes gray mold and accounts for > 30% of annual global losses valued at USD 10–100 billion (Ullah et al. 2024; Ha et al. 2021). Concurrently, horticultural efforts to recycle nutrient solutions have elevated substrate salinity, imposing abiotic stress that impairs plant physiological resilience while synergistically enhancing *B. cinerea* virulence and infectivity (Bilal et al. 2020; Islama et al. 2021; Boumaaza et al. 2015). Under saline conditions, compromised host defenses further increase disease susceptibility, with recent controlled‐environment observations confirming a synergistic incidence of salinity stress and heightened pathogen aggressiveness, presenting a critical constraint to sustainable *R. × damascena* production (Khazaeli et al. 2010). The management of *B. cinerea* presents a significant phytosanitary challenge due to its polyphagous nature, complex infection mechanisms, and adaptive reproductive strategies under variable environmental conditions (Ha et al. 2022). Although chemical fungicides remain the primary control method in *Rosa* spp. (Ha et al. 2021), their efficacy is increasingly undermined by the pathogen's genomic plasticity and rapid resistance evolution (Ha et al. 2022), alongside growing ecotoxicological and public health concerns. Concurrently, saline substrate remediation is constrained by edaphic, anthropogenic, and salt‐recalcitrance factors (Bilal et al. 2020; Islama et al. 2021), necessitating integrated, sustainable strategies to address compounding abiotic and biotic stresses exacerbated by climate variability and water scarcity.

However, current literature lacks a precise mechanistic understanding of how these concurrent stresses interact within the specific context of *R. × damascena*. While it is established that salinity alters plant water relations, ion homeostasis, redox balance, and immunity (Zhang et al. 2025), it remains unclear whether these changes primarily weaken host structural barriers or inadvertently create a more favorable microenvironment for fungal colonization. Furthermore, salinity may directly alter *B. cinerea* conidial germination and hyphal growth independent of host status, a distinction rarely disentangled in previous studies. Consequently, the interaction type, whether additive, synergistic, or antagonistic, has not been evaluated against an explicit null expectation, leading to ambiguous interpretations of “stress synergy” (Qiu et al. 2026). Recent compound‐stress literature provides a stronger conceptual framework for addressing this gap. Qiu et al. (2026) demonstrated that combined‐stress effects must be evaluated against a formal expected response derived from individual stressors, revealing that interactions may shift among multiplicative, synergistic, and antagonistic responses depending on stress intensity. Their framework separates central physiological modules such as ion homeostasis, energy metabolism, redox regulation, and threshold behavior. Similarly, Zhang et al. (2025) showed that an apparently unexpected stress interaction can only be classified after experimental separation of the underlying environmental components. Applying this framework, we hypothesize that combined salinity and *B. cinerea* infection causes injury exceeding an explicitly defined independent‐effect expectation (synergism), driven by the collapse of redox regulation and ion homeostasis.

In this context, copper‐based formulations and chitosan oligomers emerge as promising dual‐function alternatives. It is crucial to distinguish their modes of action: copper acts primarily as a direct fungicide by disrupting fungal membrane integrity and enzyme function (Rawat et al. 2024), whereas chitosan functions both as an antifungal agent via membrane permeabilization and as a potent elicitor of plant systemic acquired resistance and salinity tolerance (Hassan et al. 2021). We posit that their co‐application may restore host physiological function more strongly than expected from their individual effects, potentially through complementary pathways where copper suppresses pathogen load while chitosan reinforces host defense and osmotic adjustment. Accordingly, this study investigates the physio‐biochemical responses of *R. × damascena* to individual and combined *B. cinerea* infection and salinity stress, evaluating the interaction type against a multiplicative null model, while evaluating the therapeutic efficacy of medium‐molecular‐weight chitosan and novel nano‐chelated copper (NCC), applied singly and in combination, to mitigate these concurrent constraints.

## 2 Materials and Methods

### 2.1 Plant Selection and Cultivation Conditions

Uniform flowering specimens of *R. × damascena* were sourced from Royal Nahal Iran Nursery and selected for morphological vigor and absence of stress symptoms, with standardized dimensions (height: 50 ± 7 cm; canopy width: 30 ± 7 cm). Plants were transplanted into sterile pots containing a sterilized substrate mixture (loam 40%, decomposed bovine manure 20%, peat moss 20%, perlite 15%, vermiculite 5% v/v), with pH adjusted to 7.0 ± 0.2 using CaCO₃ or elemental sulfur; sterilization was achieved via sequential autoclaving (121°C, 15 psi, 20 min) with intermittent incubation. Following transplantation, plants were acclimatized for 13 days under controlled greenhouse conditions (30°C ± 2°C; 60% ± 5% relative humidity; 600 μmol m^−2^ s^−1^ photon flux density; 16‐h photoperiod). Irrigation was managed gravimetrically based on container water‐holding capacity (WHC), applying 50% WHC of sterile distilled water and rewatering at 25% of air‐dry mass (~1‐day interval), with uniform irrigation protocols maintained across all experimental units throughout acclimatization. Each individual pot containing a single plant was considered an independent experimental unit.

### 2.2 Application of Nano‐Chelated Copper and Chitosan

A 0.4% (w/v) stock solution of medium‐molecular‐weight chitosan (Sigma‐Aldrich, Product No. 448877; degree of deacetylation: 75%–85%; viscosity: 200–800 cP) was prepared by dissolving the polymer in dilute acetic acid (1% v/v) under continuous stirring to ensure complete solubilization at pH ~5.5. This concentration was selected based on a preliminary in vitro assay evaluating chitosan efficacy (0%–0.5% w/v) against *B. cinerea* radial growth, where 0.4% provided optimal inhibition (Figure S1). A commercial NCC formulation (Khazra; 8% w/w elemental Cu), synthesized via self‐assembly of weak organic acid ligands and nitrogen sources as described in patent US8288587B2, was used to prepare treatment solutions. (≤ 100 nm dimension; stable at pH 3–11). Treatment solutions were prepared by dissolving 1000 and 2000 mg L^−1^ of the commercial formulation in sterile distilled water, yielding nominal concentrations of 80 and 160 mg L^−1^ elemental copper, respectively. These application rates were selected to evaluate a dose–response relationship within the manufacturer's recommended range (1–2 g L^−1^).

Combined treatments were formulated by mixing appropriate volumes of the chitosan stock with the respective NCC solutions to achieve a final chitosan concentration of 0.4% (w/v). The final pH of each combined spray solution was measured to confirm physical stability and absence of precipitation. All solutions were prepared under aseptic conditions and applied immediately; chitosan‐containing mixtures were not subjected to 0.22‐μm filtration due to viscosity constraints, whereas NCC‐only solutions were filter‐sterilized (0.22 μm nylon membrane). Foliar treatments were administered on Day 13 of acclimatization using a calibrated backpack sprayer (HAIGE HG‐3WF‐3A), delivering a standardized volume of 59 mL plant^−1^ over 1.77 s to ensure uniform coverage based on mean canopy surface area (1178.1 cm^2^).

### 2.3 Simulation of Salinity

Salinity stress was induced using an equimolar mixture of analytical‐grade NaCl and CaCl_2_ (Merck KGaA, Darmstadt, Germany). This combination was selected to simulate realistic soil salinity conditions while maintaining Ca^2+^ mediated membrane stability and mitigating specific Na^+^ toxicity, as recommended for horticultural crops (Munns and Tester 2008; van Zelm et al. 2020). Two salinity regimes were applied: low salinity (LS) and high salinity (HS). The LS treatment consisted of 50 mM NaCl +50 mM CaCl_2_, yielding final ionic concentrations of 50 mM Na^+^, 50 mM Ca^2+^, and 150 mM Cl^−^, with a calculated osmotic potential of −0.37 MPa. The HS treatment consisted of 100 mM NaCl +100 mM CaCl_2_, yielding 100 mM Na^+^, 100 mM Ca^2+^, and 300 mM Cl^−^, with a calculated osmotic potential of −0.74 MPa.

To ensure precise control over pore‐water molarity, salt quantities were calculated based on substrate bulk density (0.85 g cm^−3^) and volumetric water content at 50% WHC, following established protocols for container‐grown systems (Grattan and Grieve 1998; Reis et al. 2016). Accordingly, salts were dissolved in sterile distilled water at rates of 0.73 g NaCl + 1.39 g CaCl_2_ kg^−1^ dry substrate for LS, and 1.46 g NaCl + 2.78 g CaCl_2_ kg^−1^ dry substrate for HS. These solutions were administered during the third irrigation cycle on day 14 of acclimatization, while control plants received salt‐free sterile distilled water.

### 2.4 Verification of Substrate Physicochemical Properties

Substrate electrical conductivity (EC), pH, and gravimetric moisture content were monitored in dedicated sentinel pots (*n* = 3 per salinity level) at 0, 3, and 7 days post‐salinity application. Substrate samples (10 g fresh weight) were extracted with 50 mL deionized water (1:5 w/v), shaken for 30 min, and filtered; EC and pH were measured using a calibrated multiparameter probe (Hanna HI98194). Moisture content was determined gravimetrically by drying subsamples at 105°C for 24 h. EC remained within ±10% of target values (LS: 4.2 ± 0.3 dS m^−1^; HS: 8.1 ± 0.5 dS m^−1^) across all sampling timepoints, with pH stable at 6.9–7.1 and moisture content maintained at 48%–52% WHC. Drainage fraction was not quantitatively collected; however, the high permeability of the substrate (35% perlite/vermiculite v/v) combined with strict gravimetric rewatering to 50% WHC prevented excessive salt accumulation.

### 2.5 Preparation and Inoculation of *Botrytis cinerea*

*B. cinerea* strain BS20 (GenBank Accession No. OQ318528.1), sourced from Gorgan University of Agricultural Sciences and Natural Resources (Hatamzadeh et al. 2024), was cultured on potato dextrose agar (PDA) at 24°C ± 2°C in darkness (60% relative humidity) for 5 days; conidial suspensions were prepared by eluting cultures with sterile distilled water containing 0.05% (v/v) Tween 20, filtered through Whatman No. 1 paper, and standardized to 1 × 10^4^ conidia mL^−1^ using a hemocytometer. Inoculation was performed on Day 17 of acclimatization (3 days after salinity induction) to assess the combined effect of pre‐existing salinity stress on disease susceptibility. Inoculation was performed via foliar spray (59 mL plant^−1^) using the calibrated application protocol (1.77 s plant^−1^), targeting fully expanded mature leaves and flowers, with controls receiving sterile water. Immediately following inoculation, plants were covered with transparent polyethylene bags for 48 h to maintain > 90% relative humidity and facilitate spore germination and infection. After the incubation period, bags were removed, and plants were returned to standard greenhouse conditions.

### 2.6 Experimental Design and Treatment Structure

The experiment employed a full factorial design comprising four factors: Salinity (three levels: 0, 50 + 50 mM, 100 + 100 mM NaCl+CaCl_2_), Pathogen Inoculation (two levels: Non‐inoculated, *B. cinerea* ‐inoculated), Chitosan (two levels: 0%, 0.4% w/v), and NCC (three levels: 0, 1000, 2000 mg L^−1^ formulation). This resulted in 36 unique treatment combinations (Table S1). Each treatment combination was replicated three times, resulting in a total of 108 independent experimental units (pots/plants). The three replicates per treatment represent independent biological replicates, meaning that biochemical and physiological measurements were derived from three distinct plants per treatment combination, not technical subsamples from a single plant. Measurement of plant parameters was conducted 7 days postinoculation of *B. cinerea* (at peak combined‐stress symptoms).

### 2.7 Disease Assessment

Disease severity was quantified using a standardized disease index scale ranging from 0 to 5 (Bock et al. 2022), adapted for evaluation of both leaf disease severity (LDS) and flower disease severity (FDS). The scoring criteria were defined as follows: 0 = no visible symptoms; 1 = < 10% tissue area affected (manifesting as marginal leaf necrosis or minor petal browning/wilting); 2 = 10%–25% tissue area affected (moderate leaf lesions or localized petal discoloration); 3 = 26%–50% tissue area affected (extensive leaf chlorosis/necrosis or significant floral shriveling with gray mold sporulation); 4 = 51%–75% tissue area affected (severe leaf defoliation or advanced floral maceration/collapse); and 5 = > 75% tissue area affected or complete organ death (total leaf desiccation or total floral necrosis). For each biological replicate, three leaves and three flowers per plant were evaluated independently.

### 2.8 Quantification of Photosynthetic Pigments

Photosynthetic pigments as integrative indicators of chronic photodamage were quantified via dimethyl sulfoxide (DMSO) extraction following Wellburn (1994). Briefly, fresh leaf tissue (0.05 g) was collected from each of the three independent biological replicates per treatment and incubated in 5 mL DMSO at 65°C for 30–60 min under dark conditions until complete bleaching, after which absorbance was recorded at 663, 645, and 470 nm using a UV–Vis spectrophotometer. Concentrations of total chlorophyll content (TCC) and carotenoids (CAR) were expressed as mg g^−1^ fresh weight of leaf.

### 2.9 Estimation of Proline Content

Proline accumulation (PRO) was determined spectrophotometrically using the acid‐ninhydrin method of Bates et al. (1973). Fresh leaf tissue (0.1 g) from each independent biological replicate was homogenized in 5 mL of 3% (w/v) sulfosalicylic acid and centrifuged at 10,000 *g* for 10 min at 4°C. The supernatant (2 mL) was reacted with acid‐ninhydrin reagent and glacial acetic acid, then incubated at 100°C for 60 min and rapidly cooled on ice. The proline‐ninhydrin chromophore was extracted with 4 mL toluene, and absorbance of the organic phase was measured at 520 nm. PRO was quantified against an L‐proline standard curve (0–100 μg mL^−1^) and expressed as mg g^−1^ fresh weight of leaf.

### 2.10 Assessment of Superoxide Dismutase Activity

Superoxide dismutase (SOD) activity was determined spectrophotometrically by monitoring the inhibition of nitroblue tetrazolium (NBT) photoreduction following Beauchamp and Fridovich (1971). Fresh leaf tissue (0.1 g) from each independent biological replicate was homogenized in ice‐cold 50 mM potassium phosphate buffer (pH 7.8) containing 1% (w/v) polyvinylpyrrolidone (PVP) and 0.1 mM EDTA, then centrifuged at 12,000 *g* for 15 min at 4°C to obtain the crude enzyme extract. The 3 mL reaction mixture contained 50 mM phosphate buffer (pH 7.8), 13 mM L‐methionine, 75 μM NBT, 2 μM riboflavin, 0.1 mM EDTA, and 100 μL enzyme extract, with paired non‐illuminated controls prepared for each sample. Reactions were initiated by illumination under cool‐white fluorescent lamps (Philips TL‐D 18 W/840) positioned 15 cm above the reaction tubes, providing a photosynthetic photon flux density of 45 ± 5 μmol m^−2^ s^−1^ (measured using a LI‐190R quantum sensor, LI‐COR, USA) for 10 min and terminated by extinguishing the light source; absorbance was measured at 560 nm. One unit of SOD activity was defined as the enzyme quantity causing 50% inhibition of NBT photoreduction relative to the uninhibited control, with activity expressed as units per gram fresh weight (U g^−1^).

### 2.11 Determination of Peroxidase Activity

Peroxidase (POX) activity was determined spectrophotometrically by monitoring guaiacol oxidation to tetraguaiacol following Chance and Maehly (1955). Fresh leaf tissue (0.1 g) from each independent biological replicate was homogenized in ice‐cold 50 mM potassium phosphate buffer (pH 7.0) containing 1% (w/v) PVP and centrifuged at 12,000 *g* for 15 min at 4°C to obtain the crude enzyme extract. The 3 mL reaction mixture comprised 50 mM phosphate buffer (pH 7.0), 20 mM guaiacol, 10 mM H_2_O_2_ (added last to initiate the reaction), and 100 μL enzyme extract. The increase in absorbance at 470 nm due to tetraguaiacol formation was recorded at 25°C over the initial linear phase (60–120 s), with a reagent blank included to correct for nonenzymatic oxidation. POX activity was calculated using the extinction coefficient of tetraguaiacol (ε = 26.6 mM^−1^ cm^−1^ at 470 nm) and expressed as μmol tetraguaiacol formed min^−1^ g^−1^ leaf fresh weight.

### 2.12 Measurement of Catalase Activity

Catalase (CAT) activity was determined spectrophotometrically by monitoring the decomposition of hydrogen peroxide (H_2_O_2_) at 240 nm, following the protocol of Aebi (1984). Fresh leaf tissue (0.1 g) from each independent biological replicate was homogenized in 5 mL of ice‐cold 50 mM potassium phosphate buffer (pH 7.0) containing 1% (w/v) PVP. Homogenates were centrifuged at 12,000 *g* for 15 min at 4°C, and the resulting supernatant was retained as the crude enzyme extract. The reaction mixture (3 mL) comprised 50 mM potassium phosphate buffer (pH 7.0) and 10 mM H_2_O_2_, with the reaction initiated by the addition of 100 μL enzyme extract. The decrease in absorbance at 240 nm due to H_2_O_2_ decomposition was recorded at 25°C over the initial linear phase (typically 30–60 s) using a UV–Vis spectrophotometer. A reagent blank containing all components except enzyme extract was included to correct for nonenzymatic H_2_O_2_ decay, and a sample blank (containing enzyme extract and buffer without H_2_O_2_) was measured for each sample to account for background absorbance from residual compounds in the crude extract. CAT activity was calculated using the molar extinction coefficient of H_2_O_2_ (ε = 43.6 M^−1^ cm^−1^ at 240 nm) and expressed as μmol H_2_O_2_ decomposed min^−1^ g^−1^ leaf fresh weight.

### 2.13 Assessment of Membrane Integrity via Electrolyte Leakage

Membrane stability under stress conditions was evaluated by quantifying EC following Blum and Ebercon (1981). Uniform leaf discs (0.5 cm diameter; ~0.1 g fresh weight) were excised from each of the three independent biological replicates per treatment, rinsed three times with deionized water to remove surface electrolytes and wound‐induced solutes, and blotted dry. Discs were incubated in 10 mL deionized water at 25°C ± 1°C on a rotary shaker (100 rpm) for 24 h in darkness, after which initial EC was measured at 25°C using a calibrated conductivity meter. Samples were subsequently autoclaved (121°C, 20 min) to achieve complete membrane disruption and total electrolyte release; after cooling to 25°C, final EC was recorded. A reagent blank (deionized water subjected to identical handling) was included to correct for background conductivity, and electrolyte leakage was expressed as a percentage of total cellular electrolyte content (initial EC/final EC × 100).

### 2.14 Statistical Analysis

Data were subjected to a four‐way factorial analysis of variance (ANOVA) to evaluate the main and interactive effects of salinity, *B. cinerea* inoculation, chitosan application, and NCC treatment on all measured physiological and morphological parameters. Prior to analysis, the assumptions of normality and homogeneity of variances were verified using the Shapiro–Wilk test and Levene's test, respectively. Interaction terms (Table S1) were interpreted as deviations from additive expectations, indicating nonadditive biotic–abiotic coupling or mitigation dependencies, rather than strict mathematical synergy. Means were separated using Bonferroni‐adjusted post hoc pairwise comparisons to control for Type I error across multiple comparisons. All statistical analyses were performed using JASP software (Version 0.98.1; University of Amsterdam, Netherlands). Results are presented as means ± standard error (SE), and figures were generated using Microsoft Excel (Version 2609).

## 3 Results

### 3.1 Impact of Individual Stresses on *Rosa × damascena* Physiology and Morphology

Factorial ANOVA confirmed that both salinity and pathogen stress exerted highly significant main effects across all measured parameters (Table S2). Furthermore, corresponding increases in leaf and flower disease severity were observed (Figure 1; Figure S2). Based on Bonferroni mean comparisons (Figure 2, orange indicators), under individual pathogen stress, *B. cinerea* inoculation significantly reduced CAT, POX, and SOD activities, photosynthetic pigments (TCC and CAR), and PRO levels compared to the mock control. Conversely, EC, FDS, and LDS were significantly elevated in infected plants, reflecting membrane damage and disease progression. Salinity stress induced dose‐dependent alterations in antioxidant activity and membrane integrity according to Bonferroni post hoc test (Figure 2, blue indicators). SOD and POX activities were significantly higher under 100 mM salinity compared to 50 mM and mock treatments, whereas CAT activity was significantly reduced at 100 mM relative to the mock. While TCC and CAR decreased under both salinity levels relative to the mock, the difference between 50 and 100 mM was not significant. EC increased significantly at 100 mM salinity, indicating greater electrolyte leakage. PRO accumulation was highest under 100 mM salinity, differing significantly from both 50 mM and mock treatments. FDS and LDS indices also differed significantly among salinity treatments and the mock control.

**FIGURE 1:**
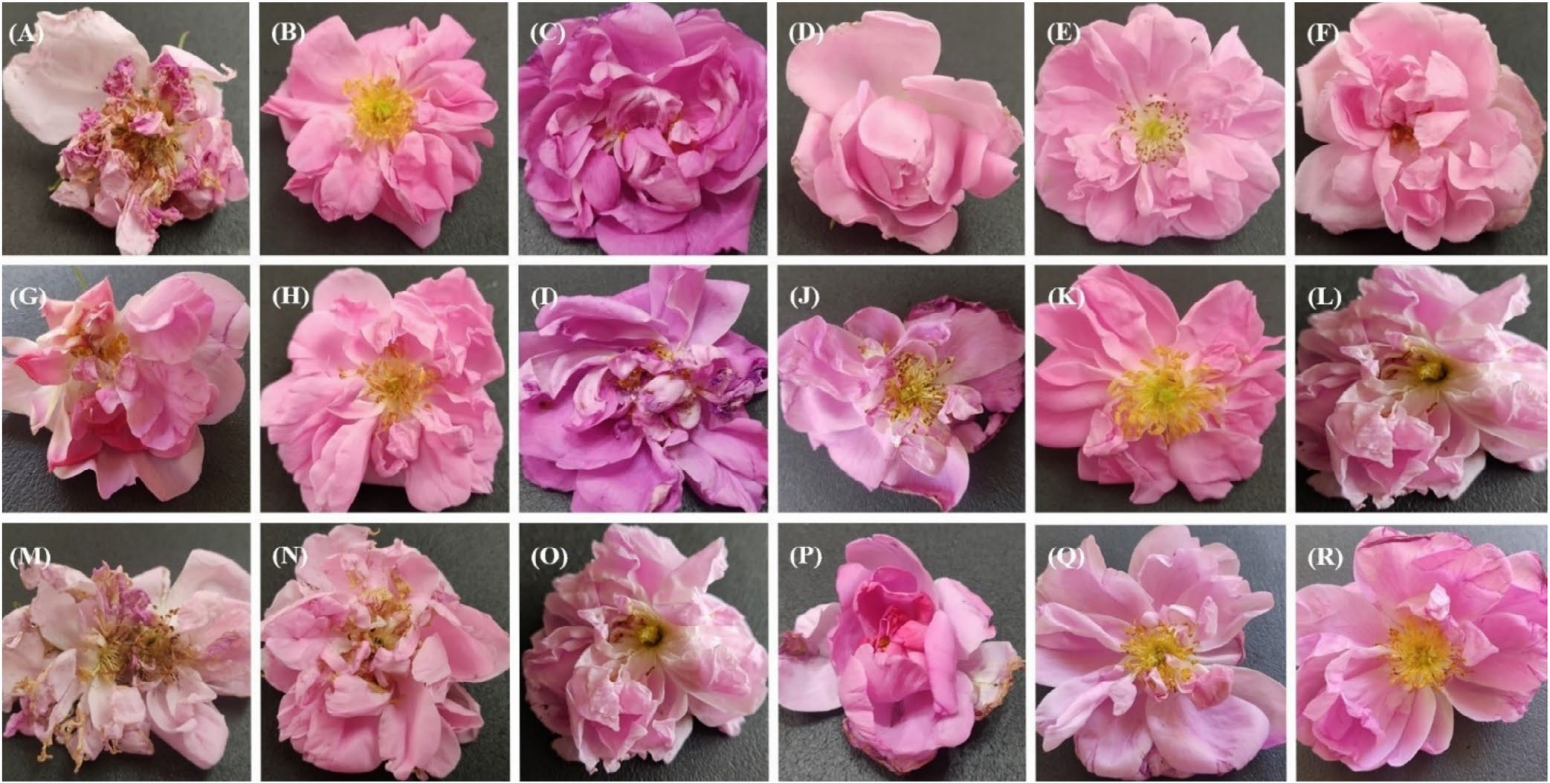
Comparative phenotypic evaluation of *Rosa × damascena* floral tissues subjected to exogenous applications of chitosan 0.4% (Ch) and nano‐chelated copper at concentrations of 1000 mg L^−1^ (NCC1) and 2000 mg L^−1^ (NCC2), under individual abiotic and biotic stress conditions: Inoculation with *Botrytis cinerea* (B.c) and salinity stress imposed at low ([LS]: 50 mM NaCl/CaCl_2_) and elevated ([HS]: 100 mM NaCl/CaCl_2_) levels. (A) B.c alone; (B) NCC1 + B.c; (C) NCC2 + B.c; (D) Ch + B.c; (E) Ch + NCC1 + B.c; (F) Ch + NCC2 + B.c; (G) LS alone; (H) NCC1 + LS; (I) NCC2 + LS; (J) Ch + LS; (K) Ch + NCC1 + LS; (L) Ch + NCC2 + LS; (M) HS alone; (N) NCC1 + HS; (O) NCC2 + HS; (P) Ch + HS; (Q) Ch + NCC1 + HS; (R) Ch + NCC2 + HS. Comparative assessment of leaf phenotypes is provided in Figure S2. Photographs taken by the authors.

**FIGURE 2:**
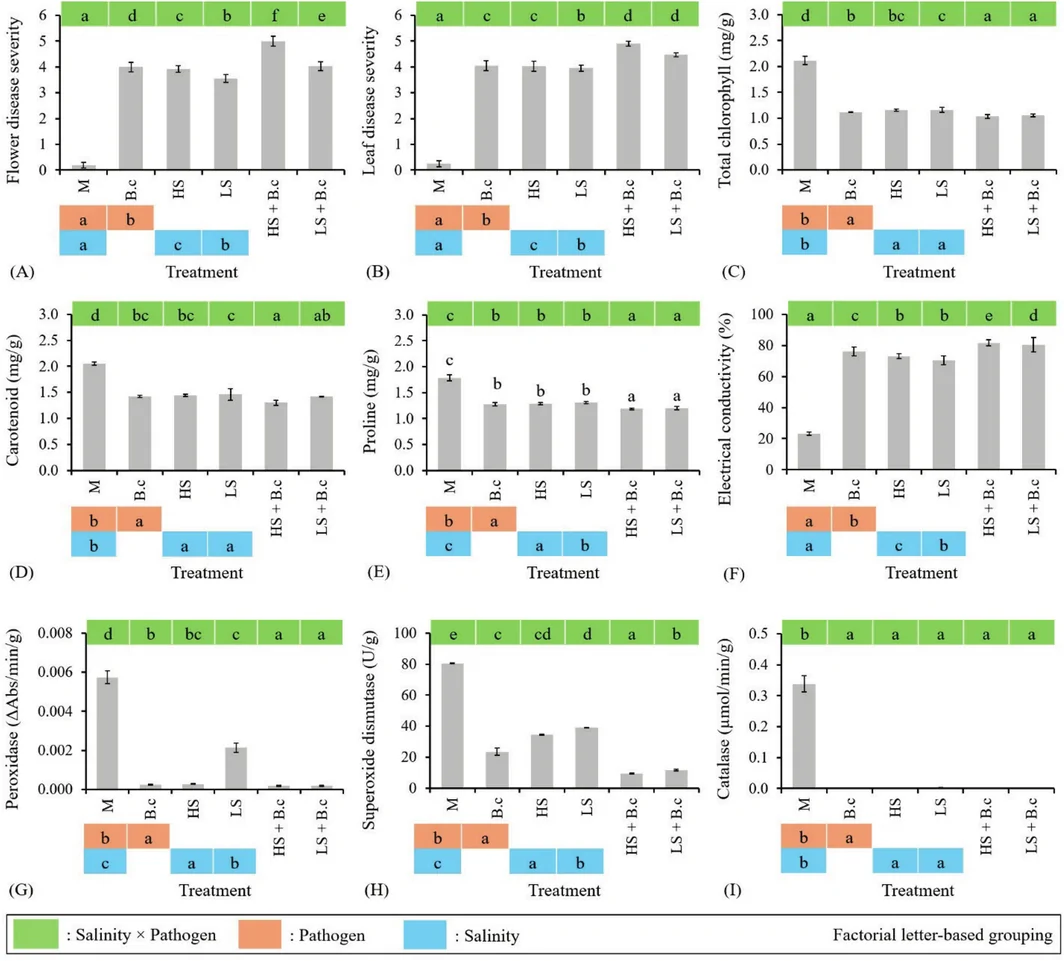
Mean comparison of physiological and morphological traits in *Rosa × damascena* under abiotic and biotic stress conditions. The panels display flower disease severity (A), leaf disease severity (B), total chlorophyll content (C), carotenoid content (D), proline content (E), electrical conductivity (F), peroxidase activity (G), superoxide dismutase activity (H), and catalase activity (I). Data are presented as means ± standard errors (SE). Treatments included a mock control (M), *Botrytis cinerea* inoculation (B.c), and salinity stress at 50 mM (LS) or 100 mM NaCl/CaCl_2_ (HS). Combined stress factors are denoted by “+”. Within each parameter, means sharing the same letter are not significantly different (Bonferroni post hoc test, *p* ≤ 0.05). Detailed factorial ANOVA results are provided in Table S2.

### 3.2 Physiological and Morphological Responses to Combined Salinity and Pathogen Stress

The interaction between salinity and pathogen stress was significant for most traits, except SOD (Table S2). Disease severity did not follow a uniform synergistic pattern (Figure 3; Figure S3). According to Bonferroni‐adjusted pairwise comparisons (Figure 2, green indicators), FDS was highest in plants subjected to *B. cinerea* under 100 mM salinity, exceeding all other treatments. However, LDS under the combined 50 mM salinity and *B. cinerea* treatment was statistically similar to the 100 mM combined stress group and significantly higher than *B. cinerea* alone, suggesting low salinity facilitates foliar infection. EC values for the 50 mM combined stress group were significantly higher than *B. cinerea* alone but distinguishable from the 100 mM combined group.

**FIGURE 3:**
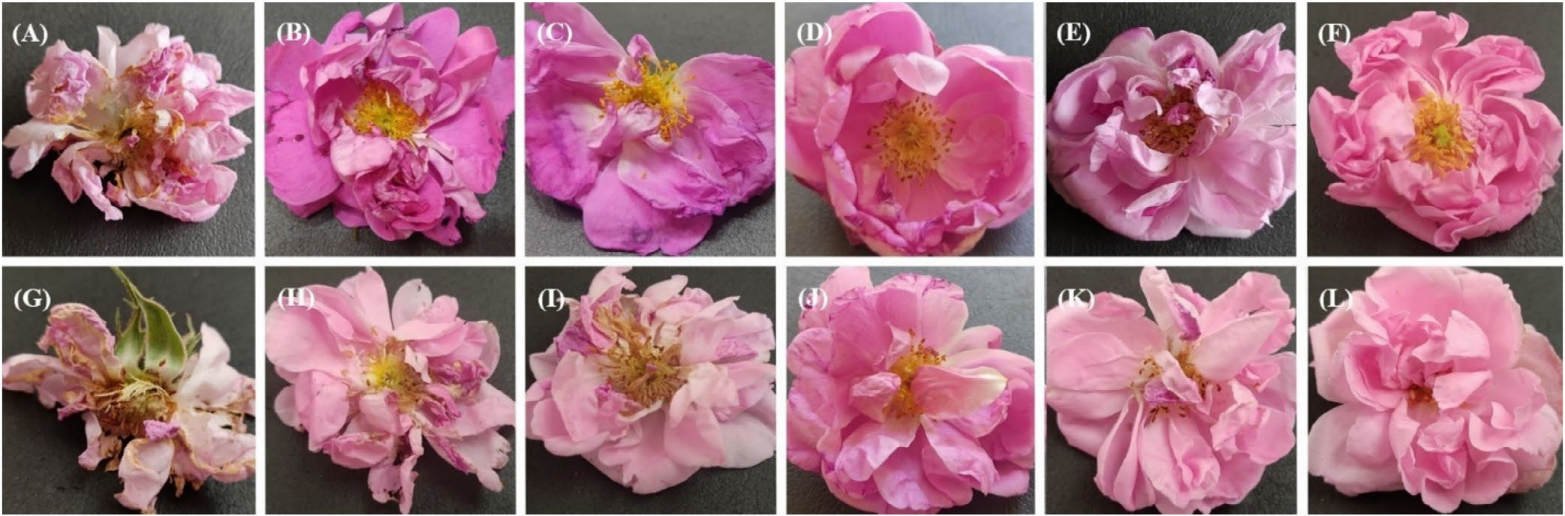
Floral morphological variations in *Rosa × damascena* subjected to combined salinity (50 mM [LS] or 100 mM [HS] NaCl/CaCl_2_) and *Botrytis cinerea* (B.c) stress, following foliar supplementation with 0.4% chitosan (Ch) and nano‐chelated copper at 1000 mg L^−1^ (NCC1) or 2000 mg L^−1^ (NCC2). Treatment groups are depicted as follows: (A) LS + B.c; (B) NCC1 + LS + B.c; (C) NCC2 + LS + B.c; (D) Ch + LS + B.c; (E) Ch + NCC1 + LS + B.c; (F) Ch + NCC2 + LS + B.c; (G) HS + B.c; (H) NCC1 + HS + B.c; (I) NCC2 + HS + B.c; (J) Ch + HS + B.c; (K) Ch + NCC1 + HS + B.c; (L) Ch + NCC2 + HS + B.c. Corresponding leaf phenotypic data are presented in Figure S3. All images presented are original photographs taken by the authors.

Antioxidant responses revealed trait‐specific interactions under combined stress. SOD activity in the 100 mM salinity + *B. cinerea* treatment was significantly lower than all other treatments, including the 50 mM combined stress group. POX activity followed a similar trend, with a significant reduction in the 100 mM combined group relative to most other treatments. CAT activity remained suppressed across all combined treatments without significant differences among them. Photosynthetic pigment degradation was most pronounced under combined conditions; TCC and CAR in both combined stress groups were significantly lower than in individual stress treatments, though they did not differ significantly from each other. PRO levels under combined stress were intermediate, being significantly lower than those observed under individual *B. cinerea* or salinity treatments.

### 3.3 Chitosan Efficacy Under Individual Stress Conditions

Factorial ANOVA for the chitosan × pathogen interaction revealed no significant effects (p>0.05) for PRO, LDS, or FDS (Table S2). As indicated by Bonferroni correction (Figure 4, blue indicators) chitosan application to *B. cinerea* ‐infected plants resulted in significantly lower POX, SOD, EC, and PRO levels compared to the infected control. Chitosan treatment maintained significantly higher TCC and CAR contents and significantly reduced LDS and FDS relative to the *B. cinerea* ‐only treatment. However, CAT activity was not significantly affected by chitosan application. However, factorial ANOVA for the chitosan × salinity interaction revealed significant differences (p≤0.05) for CAT, CAR, EC, LDS, and FDS, while other traits remained nonsignificant (Table S2). Under abiotic stress, Post hoc analysis using the Bonferroni revealed that chitosan application modulated responses to both 50 and 100 mM salinity (Figure 4; indicated by purple color). Compared to salinity‐only controls, chitosan significantly reduced EC and PRO while increasing POX and SOD activities. TCC and CAR were partially restored, and both LDS and FDS were significantly lowered. Consistent with biotic stress results, CAT activity remained unaffected by chitosan under salinity stress. These interaction effects were also evident in leaf (Figure S2) and flower (Figure 1) tissues.

**FIGURE 4:**
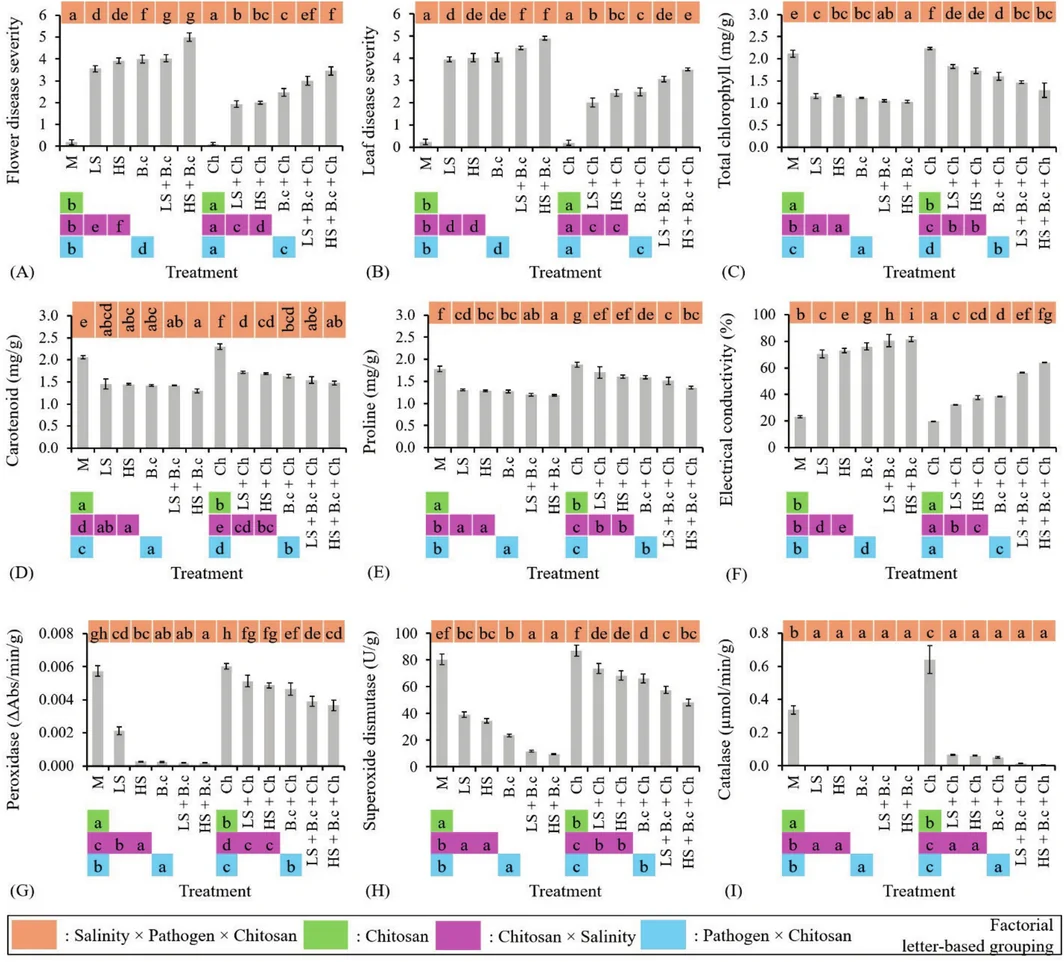
Mean comparisons of physiological and morphological parameters in *Rosa × damascena* following chitosan (0.4% w/v; [Ch]) application under isolated and concurrent stress conditions. The panels represent (A) flower disease severity, (B) leaf disease severity, (C) total chlorophyll content, (D) carotenoid content, (E) proline content, (F) electrical conductivity, (G) peroxidase activity, (H) superoxide dismutase activity, and (I) catalase activity. Data are expressed as means ± standard errors (SE). Treatments included a mock control (M), *Botrytis cinerea* inoculation (B.c), and salinity regimes of 50 mM (LS) or 100 mM NaCl/CaCl_2_ (HS), with combined factors indicated by “+”. Significant differences were determined using Bonferroni‐adjusted post hoc tests (*p* ≤ 0.05), where means sharing identical letters within each trait are statistically indistinguishable. Comprehensive ANOVA results are available in Table S2.

### 3.4 Assessment of Chitosan Application Under Combined Salinity and Fungal Stress

Significant effects of the salinity × pathogen × chitosan interaction were observed for POX, CAT, EC, LDS, and FDS (p≤0.05); no significant differences were detected for the remaining parameters (Table S2). Supported by Bonferroni‐derived significance tests (Figure 4, orange indicators), chitosan application significantly reduced EC and increased PRO and SOD levels under combined moderate stress (50 mM salinity + *B. cinerea* ), relative to the untreated combined stress group. However, POX, CAT, TCC, and CAR did not differ significantly between chitosan‐treated and untreated plants under this condition. Morphologically, chitosan significantly reduced both LDS and FDS. Under severe combined stress (100 mM salinity + *B. cinerea* ), chitosan elicited a similar response, with significant reductions in EC and increases in SOD. A notable exception was TCC, which differed significantly between chitosan‐treated and untreated plants under severe stress. As with moderate stress, POX, CAT, and CAR showed no significant differences between treated and untreated groups, while LDS and FDS were significantly reduced by chitosan application. The impact of this interaction was also morphologically apparent in both foliage (Figure S3) and floral (Figure 3) tissues.

### 3.5 Mitigation of Individual Stressors by Nano‐Chelated Copper

Statistical analysis of the pathogen × NCC interaction identified CAT and SOD as the only parameters exhibiting significant variation among treatments (Table S2) As indicated by Bonferroni‐corrected pairwise tests (Figure 5, blue indicators), application of NCC (1000 and 2000 mg L^−1^) to *B. cinerea* ‐inoculated plants significantly reduced EC and increased PRO and TCC relative to the untreated infected group. POX and SOD activities were significantly higher in NCC‐treated plants, while CAT and CAR levels did not differ significantly from the infected control. Both NCC concentrations significantly reduced LDS and FDS compared to the *B. cinerea* ‐only treatment.

**FIGURE 5:**
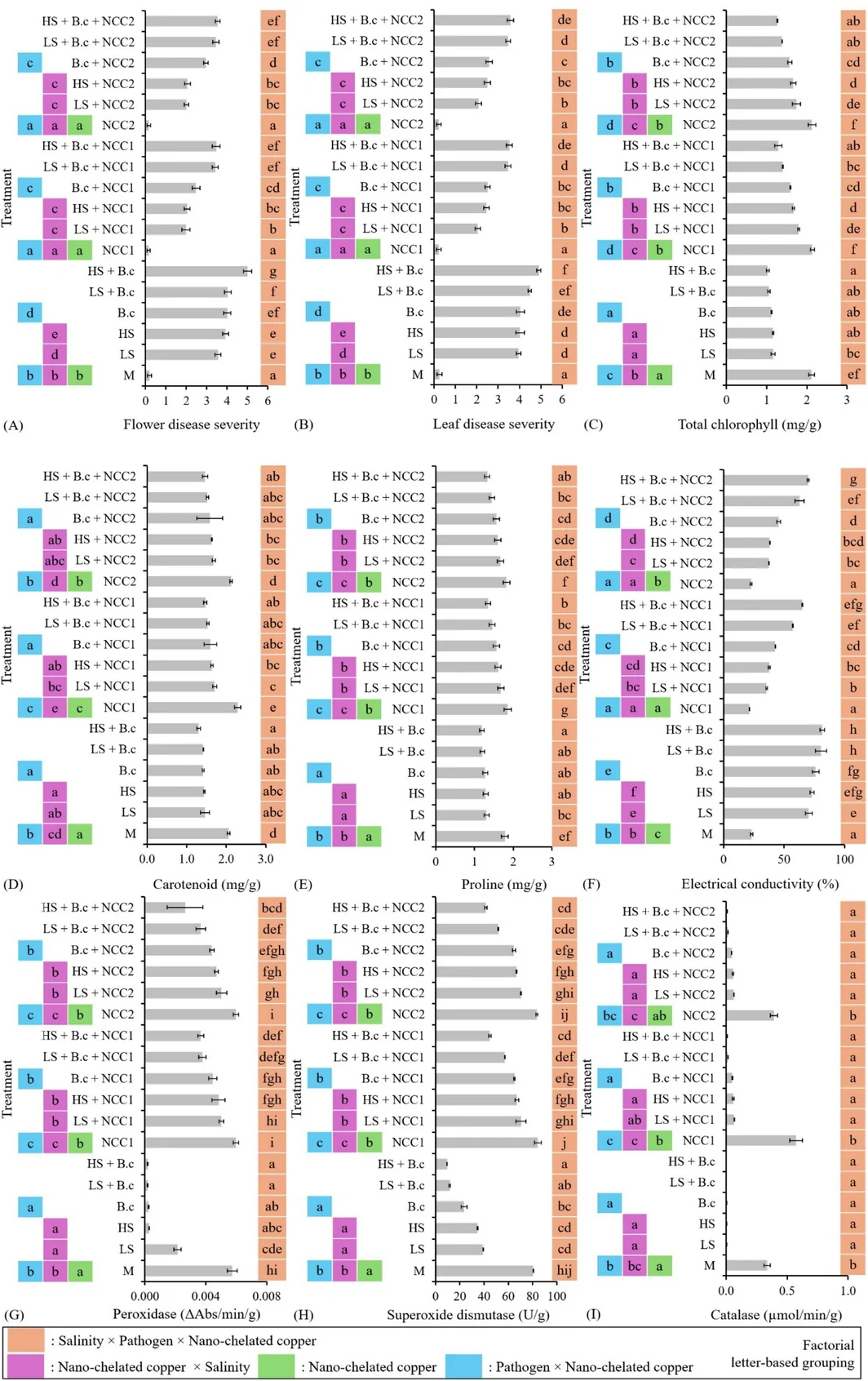
Mean values of physiological and morphological traits in *Rosa × damascena* treated with nano‐chelated copper (1000 mg L^−1^ [NCC1] or 2000 mg L^−1^ [NCC2]) under biotic (*Botrytis cinerea*; [B.c]) and abiotic (50 mM [LS] or 100 mM [HS] NaCl/CaCl_2_) stress. The panels illustrate (A) flower disease severity, (B) leaf disease severity, (C) total chlorophyll content, (D) carotenoid content, (E) proline content, (F) electrical conductivity, (G) peroxidase activity, (H) superoxide dismutase activity, and (I) catalase activity. Error bars represent standard errors (SE). Treatments include a mock control (M) and combined applications denoted by “+”. Statistical significance was assessed via Bonferroni‐adjusted post hoc comparisons (*p* ≤ 0.05), with means sharing the same letter indicating no significant difference. Detailed ANOVA statistics are provided in Table S2.

At the 0.05 significance level, the salinity × NCC interaction exerted a statistically significant influence on CAR, EC, LDS, and FDS, while no such effects were observed for the remaining parameters (Table S2). Derived from Bonferroni mean comparisons (Figure 5, purple indicators), NCC application at both concentrations significantly lowered EC and increased PRO and TCC under salinity stress relative to salinity‐only controls. POX and SOD activities were significantly enhanced by NCC, whereas CAT and CAR remained statistically similar to untreated salinity controls. Morphologically, NCC significantly reduced both LDS and FDS under 50 and 100 mM salinity conditions. These effects (pathogen or salinity × NCC interactions) were also observed in leaf and flower symptomatology (Figure 1; Figure S2).

### 3.6 Potential of Nano‐Chelated Copper in Alleviating the Adverse Effects of Combined Stresses

Among the measured parameters, POX, SOD, CAR, EC, LDS, and FDS were significantly responsive to the pathogen × NCC × salinity interaction, whereas the remaining traits displayed no statistically significant variation (Table S2). As shown in Bonferroni‐derived significance tests (Figure 5, orange indicators), under moderate combined stress (50 mM salinity + *B. cinerea* ), NCC application (1000 or 2000 mg L^−1^) significantly reduced EC, POX, and SOD relative to the untreated group. TCC, CAR, PRO, and CAT did not differ significantly between NCC‐treated and untreated plants. Morphologically, NCC significantly reduced LDS, but FDS remained statistically similar to the untreated combined stress treatment. Under severe combined stress (100 mM salinity + *B. cinerea* ), NCC application resulted in significantly higher POX and SOD activities and lower EC compared to the untreated group. CAT, TCC, and CAR did not differ significantly between treatments. Both NCC concentrations significantly reduced LDS and FDS. Specifically, the 1000 mg L^−1^ NCC treatment resulted in significantly lower PRO levels compared to the untreated severe combined stress group, whereas the 2000 mg L^−1^ treatment did not differ significantly from the control. Corroborating this interaction effect, distinct alterations in leaf and flower symptomatology were evident (Figure 3; Figure S3).

### 3.7 Combined Application of Chitosan and Nano‐Chelated Copper Under Individual Stress Conditions

Significant variation attributable to the pathogen × NCC × chitosan interaction was restricted to SOD and CAR, with all others remaining statistically unaffected (Table S2). In accordance with Bonferroni mean comparations (Tables 1, 2, 3, 4, “●” symbol), under *B. cinerea* infection, combination treatments (1000 or 2000 mg L^−1^ NCC + chitosan) resulted in significantly higher POX and SOD activities and lower EC relative to the infected control. TCC was significantly higher in both combination treatments, while CAR increased significantly only at the 1000 mg L^−1^ NCC concentration. PRO levels differed significantly between combination treatments and the untreated infected group, while CAT remained similar across all treatments. Both combinations significantly reduced LDS and FDS.

**TABLE 1:** Comparative mean values for disease severity observed in *Rosa* × *damascena* across four experimental configurations of complete factorial (▲), salinity × nano‐chelated copper × chitosan (**■**), pathogen × nano‐chelated copper × chitosan (**●**), and nano‐chelated copper × chitosan (**♦**).

Treatment	Flower disease severity					Leaf disease severity				
	Mean ± SE	▲	■	●	♦	Mean ± SE	▲	■	●	♦
M	0.19 ± 0.10	ab	ef	c	c	0.25 ± 0.12	abc	cd	cd	c
LS	3.55 ± 0.14	hi	h			3.95 ± 0.11	ij	g		
HS	3.92 ± 0.13	i	i			4.03 ± 0.2	ij	g		
B.c	4 ± 0.18	i		e		4.05 ± 0.19	ij		f	
LS + B.c	4.03 ± 0.17	i				4.48 ± 0.08	jk			
HS + B.c	5 ± 0.19	j				4.9 ± 0.09	k			
Ch	0.11 ± 0.06	a	bc	b	b	0.19 ± 0.11	abc	b	b	b
LS + Ch	1.94 ± 0.15	ef	fg			2.01 ± 0.21	ef	def		
HS + Ch	2.00 ± 0.06	ef	g			2.45 ± 0.15	fg	f		
B.c + Ch	2.47 ± 0.17	fg		d		2.5 ± 0.17	fg		de	
LS + B.c + Ch	3 ± 0.20	gh				3.07 ± 0.12	gh			
HS + B.c + Ch	3.45 ± 0.19	hi				3.5 ± 0.06	hi			
NCC1	0.15 ± 0.07	a	c	b	b	0.21 ± 0.11	abc	b	b	b
LS + NCC1	1.98 ± 0.19	ef	g			2.04 ± 0.13	ef	ef		
HS + NCC1	2.04 ± 0.14	ef	g			2.45 ± 0.13	fg	f		
B.c + NCC1	2.47 ± 0.18	fg		d		2.5 ± 0.12	fg		e	
LS + B.c + NCC1	3.41 ± 0.14	hi				3.46 ± 0.15	hi			
HS + B.c + NCC1	3.46 ± 0.18	hi				3.52 ± 0.14	hi			
NCC1 + Ch	0.08 ± 0.04	a	a	a	a	0.08 ± 0.04	a	a	a	a
LS + Ch + NCC1	0.45 ± 0.18	abc	cde			0.96 ± 0.1	bcd	c		
HS + Ch + NCC1	0.98 ± 0.08	bcd	def			1.02 ± 0.2	cd	cd		
B.c + Ch + NNC1	1.01 ± 0.08	cd		c		1.5 ± 0.21	de		c	
LS + B.c + Ch + NCC1	2.96 ± 0.15	gh				2.99 ± 0.21	gh			
HS + B.c + Ch + NCC1	2.98 ± 0.08	gh				3.02 ± 0.16	gh			
NCC2	0.16 ± 0.09	a	cd	b	b	0.21 ± 0.11	abc	b	b	b
LS + NCC2	2 ± 0.10	ef	g			2.08 ± 0.15	ef	f		
HS + NCC2	2.05 ± 0.14	ef	g			2.48 ± 0.15	fg	f		
B.c + NCC2	2.95 ± 0.11	gh		d		2.57 ± 0.15	fg		e	
LS + B.c + NCC2	3.44 ± 0.16	hi				3.47 ± 0.11	hi			
HS + B.c + NCC2	3.53 ± 0.11	hi				3.57 ± 0.15	hi			
NCC2 + Ch	0.08 ± 0.04	a	ab	a	a	0.14 ± 0.07	ab	ab	a	a
LS + Ch + NCC2	0.48 ± 0.16	abc	cde			1.02 ± 0.17	cd	cd		
HS + Ch + NCC2	1 ± 0.08	cd	def			1.45 ± 0.18	de	cde		
B.c + Ch + NNC2	1.47 ± 0.13	de		c		1.96 ± 0.13	ef		cd	
LS + B.c + Ch + NCC2	2.98 ± 0.18	gh				3 ± 0.23	gh			
HS + B.c + Ch + NCC2	3 ± 0.13	gh				3.02 ± 0.18	gh			

*Note:* The study employed a range of treatments: a control group (M), *Botrytis cinerea* inoculation (B.c), saline conditions at 50 mM (LS) and 100 mM NaCl/CaCl_2_ (HS), and foliar doses of nano‐chelated copper (1000 mg L^−1^ [NCC1], 2000 mg L^−1^ [NCC2]) and chitosan (0.4% w/v [Ch]). Interactions between these factors are represented by “+”. Statistical homogeneity is indicated by matching letters within columns. Comprehensive ANOVA outputs for the factorial experiment are summarized in Table S2.

**TABLE 2:** Assessment of mean differences among treatments across four experimental configurations of complete factorial (▲), salinity × nano‐chelated copper × chitosan (**■**), pathogen × nano‐chelated copper × chitosan (**●**), and nano‐chelated copper × chitosan (**♦**) related to leaf pigments in *Rosa* × *damascena*.

Treatment	Total chlorophyll (mg/g)					Carotenoid (mg/g)				
	Mean ± SE	▲	■	●	♦	Mean ± SE	▲	■	●	♦
M	2.12 ± 0.08	klmno	bcde	bc	a	2.06 ± 0.03	defg	bcde	bcd	a
LS	1.16 ± 0.05	abcd	a			1.46 ± 0.11	ab	ab		
HS	1.16 ± 0.02	abcd	a			1.44 ± 0.02	ab	a		
B.c	1.12 ± 0.01	abc		a		1.42 ± 0.02	ab		a	
LS + B.c	1.06 ± 0.03	ab				1.42 ± 0.01	ab			
HS + B.c	1.03 ± 0.03	a				1.3 ± 0.05	a			
Ch	2.24 ± 0.03	no	f	f	b	2.3 ± 0.06	fg	ef	e	bc
LS + Ch	1.83 ± 0.05	ghijklmn	bcdef			1.72 ± 0.03	abcde	abcd		
HS + Ch	1.73 ± 0.07	efghijklm	bc			1.68 ± 0.02	abcde	abc		
B.c + Ch	1.61 ± 0.09	defghij		bc		1.63 ± 0.03	abcde		ab	
LS + B.c + Ch	1.47 ± 0.03	abcdefgh				1.54 ± 0.08	abc			
HS + B.c + Ch	1.29 ± 0.16	abcdef				1.47 ± 0.05	ab			
NCC1	2.14 ± 0.05	mno	ef	ef	b	2.29 ± 0.08	fg	def	de	bc
LS + NCC1	1.8 ± 0.02	ghijklmn	bcde			1.71 ± 0.05	abcde	abc		
HS + NCC1	1.67 ± 0.03	efghijkl	b			1.64 ± 0.03	abcde	abc		
B.c + NCC1	1.59 ± 0.01	defghi		b		1.6 ± 0.16	abcd		ab	
LS + B.c + NCC1	1.4 ± 0.01	abcdefg				1.54 ± 0.03	abc			
HS + B.c + NCC1	1.29 ± 0.09	abcdef				1.47 ± 0.04	ab			
NCC1 + Ch	2.77 ± 0.06	p	g	g	c	3.44 ± 0.05	h	g	f	d
LS + Ch + NCC1	2.08 ± 0.01	klmn	def			2 ± 0.06	cdefg	cdef		
HS + Ch + NCC1	2.07 ± 0.09	jklmn	cdef			1.83 ± 0.03	bcdef	abcde		
B.c + Ch + NNC1	1.93 ± 0.03	hijklmn		cde		1.77 ± 0.03	abcde		bcd	
LS + B.c + Ch + NCC1	1.53 ± 0.06	cdefgh				1.58 ± 0.04	abcd			
HS + B.c + Ch + NCC1	1.5 ± 0.2	bcdefgh				1.55 ± 0.01	abc			
NCC2	2.13 ± 0.09	lmno	def	def	b	2.12 ± 0.02	efg	cdef	cde	b
LS + NCC2	1.74 ± 0.1	fghijklm	bcd			1.68 ± 0.04	abcde	abc		
HS + NCC2	1.66 ± 0.07	efghijk	b			1.64 ± 0.01	abcde	abc		
B.c + NCC2	1.57 ± 0.05	cdefghi		b		1.59 ± 0.33	abcd		ab	
LS + B.c + NCC2	1.38 ± 0.01	abcdefg				1.52 ± 0.03	abc			
HS + B.c + NCC2	1.27 ± 0.01	abcde				1.46 ± 0.07	ab			
NCC2 + Ch	2.57 ± 0.06	op	g	g	c	2.39 ± 0.27	g	f	e	c
LS + Ch + NCC2	2.07 ± 0.06	klmn	cdef			1.85 ± 0.03	bcdef	bcde		
HS + Ch + NCC2	2 ± 0.03	ijklmn	bcdef			1.83 ± 0.02	bcdef	abcde		
B.c + Ch + NNC2	1.91 ± 0.17	hijklmn		bcd		1.75 ± 0.03	abcde		abc	
LS + B.c + Ch + NCC2	1.5 ± 0.23	bcdefgh				1.57 ± 0.01	abcd			
HS + B.c + Ch + NCC2	1.48 ± 0.02	abcdefgh				1.55 ± 0.13	abc			

*Note:* Plants were subjected to the following conditions: mock treatment (M), *Botrytis cinerea* inoculation (B.c), salinity at 50 mM (LS) or 100 mM NaCl/CaCl_2_ (HS), and foliar applications of nano‐chelated copper at concentrations of 1000 mg L^−1^ (NCC1) and 2000 mg L^−1^ (NCC2), alongside chitosan at 0.4% w/v (Ch). Combinatorial applications are signified by “+”. Within each column, means sharing the same letter belong to homogeneous statistical subsets. Full ANOVA results for the factorial design are available in Table S2.

**TABLE 3:** Illustration the statistical comparison of electrical conductivity and Proline accumulation means in *Rosa* × *damascena* across configurations of complete factorial (▲), salinity × nano‐chelated copper × chitosan (**■**), pathogen × nano‐chelated copper × chitosan (**●**), and nano‐chelated copper × chitosan (♦).

Treatment	Electrical conductivity (%)					Proline (mg/g)				
	Mean ± SE	▲	■	●	♦	Mean ± SE	▲	■	●	♦
M	23.19 ± 1	abcd	ghi	de	d	1.78 ± 0.06	ijklm	bcde	b	a
LS	70.48 ± 2.79	pqr	j			1.31 ± 0.02	abcd	a		
HS	73.06 ± 1.61	qrs	j			1.29 ± 0.02	abc	a		
B.c	76.12 ± 2.86	rs		f		1.28 ± 0.03	ab		a	
LS + B.c	80.56 ± 4.72	s				1.2 ± 0.03	a			
HS + B.c	81.6 ± 1.92	s				1.19 ± 0.02	a			
Ch	19.77 ± 0.19	ab	bc	b	b	1.88 ± 0.06	lm	fg	de	b
LS + Ch	32.38 ± 0.04	efgh	fg			1.7 ± 0.13	ghijklm	bcdef		
HS + Ch	37.53 ± 1.46	ghij	hi			1.61 ± 0.04	fghijkl	bcd		
B.c + Ch	38.6 ± 0.09	hijkl		d		1.59 ± 0.04	efghijk		bc	
LS + B.c + Ch	56.35 ± 0.15	no				1.51 ± 0.08	bcdefghi			
HS + B.c + Ch	63.99 ± 0.28	op				1.36 ± 0.03	abcdef			
NCC1	21.2 ± 0.02	abc	cd	b	b	1.85 ± 0.03	klm	fg	de	b
LS + NCC1	35.6 ± 0.6	fghi	gh			1.68 ± 0.08	ghijklm	bcdef		
HS + NCC1	37.76 ± 0.78	ghij	hi			1.61 ± 0.04	fghijkl	bc		
B.c + NCC1	42.67 ± 0.13	ijklm		d		1.57 ± 0.01	defghij		b	
LS + B.c + NCC1	57.15 ± 0.25	no				1.46 ± 0.01	abcdefgh			
HS + B.c + NCC1	65.12 ± 0.32	opq				1.35 ± 0.02	abcdef			
NCC1 + Ch	14.55 ± 0.18	a	a	a	a	2.26 ± 0.09	n	h	f	c
LS + Ch + NCC1	24.9 ± 0.41	bcde	de			1.76 ± 0.02	ijklm	defg		
HS + Ch + NCC1	26.47 ± 2.2	bcde	de			1.74 ± 0.01	ijklm	cdefg		
B.c + Ch + NNC1	29.23 ± 0.34	cdefg		c		1.73 ± 0.03	hijklm		cd	
LS + B.c + Ch + NCC1	46.68 ± 1.53	klm				1.56 ± 0.06	cdefghij			
HS + B.c + Ch + NCC1	47.15 ± 3.79	lm				1.56 ± 0.04	cdefghij			
NCC2	22.77 ± 0.62	abcd	cde	b	c	1.83 ± 0.03	jklm	efg	de	b
LS + NCC2	37.36 ± 0.14	ghij	ghi			1.67 ± 0.03	ghijklm	bcdef		
HS + NCC2	38.11 ± 0.18	ghijk	i			1.6 ± 0.01	efghijk	b		
B.c + NCC2	45.58 ± 1.43	jklm		e		1.57 ± 0.01	defghij		b	
LS + B.c + NCC2	62.72 ± 3.68	op				1.45 ± 0.02	abcdefg			
HS + B.c + NCC2	69.8 ± 0.71	pqr				1.33 ± 0.06	abcde			
NCC2 + Ch	15.85 ± 0.37	a	ab	a	a	1.91 ± 0.09	m	g	ef	c
LS + Ch + NCC2	25.33 ± 0.1	bcde	de			1.75 ± 0.02	ijklm	defg		
HS + Ch + NCC2	28.18 ± 1.01	bcdef	ef			1.73 ± 0.02	hijklm	bcdef		
B.c + Ch + NNC2	31.3 ± 0.08	defgh		c		1.73 ± 0.02	hijklm		cd	
LS + B.c + Ch + NCC2	46.88 ± 0.71	klm				1.56 ± 0.06	cdefghij			
HS + B.c + Ch + NCC2	51.36 ± 0.05	mn				1.53 ± 0.06	bcdefghi			

*Note:* Treatment variables comprised a non‐treated control (M), *Botrytis cinerea* infection (B.c), two levels of salt stress 50 mM (LS) and 100 mM NaCl/CaCl_2_ (HS) and foliar sprays of nano‐chelated copper (1000 mg L^−1^ [NCC1]; 2000 mg L^−1^ [NCC2]) and chitosan (0.4% w/v [Ch]). Combined treatments are denoted by “+”. Statistically similar means within each column are marked by identical letters. The complete factorial ANOVA data are listed in Table S2.

For the salinity × NCC × chitosan interaction, SOD, FDS, and CAT were the only parameters that remained statistically nonsignificant, while all other traits exhibited significant variation (p≤0.05). Bonferroni post hoc analysis (Tables 1, 2, 3, 4, “■” symbol) revealed that under low salinity, combination treatments significantly enhanced POX, SOD, PRO, and TCC levels while concurrently reducing EC relative to the untreated control. CAR was significantly higher only in the 2000 mg L^−1^ NCC + chitosan treatment. CAT activity remained statistically similar across treatments. Both combinations significantly reduced LDS and FDS. Under severe salinity, combination treatments similarly improved membrane integrity (lower EC) and pigment retention (higher TCC). POX and SOD differed significantly between combination‐treated and untreated plants, while CAT and CAR remained similar. PRO was significantly higher in both combination treatments under severe stress. Despite biochemical variations, both formulations significantly reduced LDS and FDS. These interaction effects were further reflected in the morphological status of foliage and floral tissues, as depicted in Figure 1 and Figure S2.

**TABLE 4:** Mean comparison analysis of enzymatic activity in *Rosa* × *damascena* across full (**▲**), salinity × nano‐chelated copper × chitosan (**■**), pathogen × nano‐chelated copper × chitosan (**●**), and nano‐chelated copper × chitosan (**♦**) factorial designs.

Treatment	Peroxidase (ΔAbs/min/g)					Superoxide dismutase (U/g)					Catalase (μmol/min/g)				
	Mean ± SE	▲	■	●	♦	Mean ± SE	▲	■	●	♦	Mean ± SE	▲	■	●	♦
M	0.0057 ± 0.0003	fgh	b	b	a	80.49 ± 0.09	klmno	b	b	a	0.338 ± 0.256	ab	abc	abcd	a
LS	0.0021 ± 0.0002	b	a			39.13 ± 0.07	bcd	a			0.004 ± 0.0001	a	a		
HS	0.0003 ± 0.0001	a	a			34.61 ± 0.26	bc	a			0.002 ± 0	a	a		
B.c	0.0002 ± 0.0001	a		a		23.5 ± 2.31	ab		a		0.001 ± 0.0001	a		a	
LS + B.c	0.0002 ± 0.0001	a				11.63 ± 0.49	a				0.0005 ± 0.0003	a			
HS + B.c	0.0002 ± 0.0001	a				9.43 ± 0.31	a				0.0002 ± 0.0001	a			
Ch	0.006 ± 0.0002	fgh	def	ef	bc	87 ± 0.2	mno	ghi	ef	b	0.64 ± 0.085	bcd	cd	cde	ab
LS + Ch	0.0051 ± 0.0003	defgh	cd			73.55 ± 0.52	hijklmn	cdefg			0.068 ± 0.003	a	ab		
HS + Ch	0.0049 ± 0.0001	def	cd			68.46 ± 0.74	fghijklmn	bcde			0.061 ± 0.001	a	ab		
B.c + Ch	0.0046 ± 0.0004	def		cd		66.1 ± 1.5	fghijkl		bc		0.051 ± 0.005	a		ab	
LS + B.c + Ch	0.0039 ± 0.0003	bcde				57.62 ± 0.27	defghi				0.015 ± 0.001	a			
HS + B.c + Ch	0.0037 ± 0.0003	bcd				48.29 ± 20.48	cdef				0.007 ± 0.0001	a			
NCC1	0.006 ± 0.0002	fgh	def	ef	b	84.28 ± 2.8	lmno	ghi	de	b	0.573 ± 0.052	bcd	bcd	bcde	ab
LS + NCC1	0.005 ± 0.0001	defg	cd			70.5 ± 4.04	ghijklmn	bcdefg			0.067 ± 0.001	a	ab		
HS + NCC1	0.0048 ± 0.0004	def	cd			67.05 ± 1.36	fghijklm	bcd			0.057 ± 0.008	a	ab		
B.c + NCC1	0.0045 ± 0.0003	def		cd		65 ± 0.53	efghijkl		b		0.051 ± 0.003	a		ab	
LS + B.c + NCC1	0.0038 ± 0.0002	bcde				57.02 ± 0.2	defgh				0.013 ± 0.001	a			
HS + B.c + NCC1	0.0037 ± 0.0002	bcd				44.83 ± 0.81	cde				0.007 ± 0.001	a			
NCC1 + Ch	0.0069 ± 0.0002	h	f	f	d	94.75 ± 0.8	o	i	f	c	0.927 ± 0.029	d	d	e	b
LS + Ch + NCC1	0.0056 ± 0.0002	efgh	def			79.4 ± 0.14	jklmno	fgh			0.325 ± 0.233	ab	abc		
HS + Ch + NCC1	0.0054 ± 0.0003	defgh	de			77.82 ± 0.54	ijklmno	defg			0.076 ± 0.008	a	ab		
B.c + Ch + NNC1	0.0052 ± 0.0002	defgh		de		75.64 ± 1.78	hijklmno		cd		0.071 ± 0.004	a		abc	
LS + B.c + Ch + NCC1	0.0044 ± 0.0003	cdef				63.92 ± 2.54	efghijkl				0.045 ± 0.002	a			
HS + B.c + Ch + NCC1	0.0043 ± 0.0003	cdef				59.05 ± 0.04	defghij				0.03 ± 0.001	a			
NCC2	0.006 ± 0.0002	fgh	def	ef	b	83.43 ± 0.83	klmno	fghi	de	b	0.392 ± 0.309	abc	abcd	abcd	a
LS + NCC2	0.005 ± 0.0004	defg	cd			70.4 ± 0.4	ghijklmn	bcdef			0.062 ± 0.002	a	ab		
HS + NCC2	0.0047 ± 0.0001	def	bc			66.72 ± 0.36	fghijklm	bc			0.057 ± 0.006	a	ab		
B.c + NCC2	0.0044 ± 0.0002	cdef		bc		64.57 ± 1.39	efghijkl		b		0.045 ± 0.003	a		ab	
LS + B.c + NCC2	0.0037 ± 0.0003	bcd				51.82 ± 0.48	cdefg				0.013 ± 0.001	a			
HS + B.c + NCC2	0.0026 ± 0.0012	bc				41.37 ± 0.92	bcd				0.007 ± 0.0001	a			
NCC2 + Ch	0.0068 ± 0.0002	gh	ef	f	cd	88.98 ± 0.32	no	hi	ef	c	0.84 ± 0.046	cd	cd	de	ab
LS + Ch + NCC2	0.0054 ± 0.0004	defgh	de			77.9 ± 0.64	ijklmno	efgh			0.077 ± 0.002	a	ab		
HS + Ch + NCC2	0.0053 ± 0.0003	defgh	cd			77.56 ± 0.32	hijklmno	defg			0.073 ± 0.002	a	ab		
B.c + Ch + NNC2	0.0052 ± 0.0001	defgh		de		75.21 ± 2.08	hijklmno		cd		0.069 ± 0.006	a		abc	
LS + B.c + Ch + NCC2	0.0043 ± 0.0002	cdef				63.15 ± 1	efghijk				0.034 ± 0.002	a			
HS + B.c + Ch + NCC2	0.0042 ± 0.0004	cdef				58.82 ± 0.24	defghij				0.024 ± 0.002	a			

*Note:* The experimental treatments included a mock control (M), inoculation with *Botrytis cinerea* (B.c), salinity stress induced by 50 mM (LS) or 100 mM NaCl/CaCl_2_ (HS), and foliar applications of nano‐chelated copper at 1000 mg L^−1^ (NCC1) or 2000 mg L^−1^ (NCC2), as well as chitosan at 0.4% w/v (Ch). Various combinations of these factors were also evaluated, indicated by a “+” symbol. Columns featuring different letters represent groups that are not statistically distinct. Detailed results from the factorial ANOVA are provided in Table S2.

### 3.8 Combination of Chitosan and Nano‐Chelated Copper in Mitigating Combined Stress Conditions

Statistical analysis of the four‐way interaction (salinity × NCC × chitosan × pathogen) identified POX, PRO, EC, and CAR as the only significantly responsive parameters (Table S2). Subsequent Bonferroni post hoc comparisons (Tables 1, 2, 3, 4, “▲” symbol) demonstrated that under moderate combined stress, co‐application of NCC and chitosan significantly elevated POX and SOD activities while reducing EC relative to the untreated control. TCC was significantly higher only in the 1000 mg L^−1^ NCC + chitosan treatment, while CAR and CAT did not differ significantly. PRO levels varied significantly across all groups. Morphologically, both formulations significantly reduced LDS and FDS. Under severe combined stress, co‐application of chitosan and NCC (1000 or 2000 mg L^−1^) resulted in significantly lower EC and higher POX, SOD, and TCC compared to the untreated severe combined stress group. CAR and CAT did not differ significantly between treatments. PRO levels varied significantly across groups. Both formulations significantly reduced LDS and FDS relative to plants subjected to severe combined stress without supplementation. Visual assessment of plant morphology confirmed these interaction effects, with notable changes in leaf and flower symptomatology documented in Figure 3 and Figure S3.

### 3.9 Chitosan and Nano‐Chelated Copper as Nontoxic Factors Against Rose Plant

In the absence of stress, while main effects for both chitosan or NCC were significant across all measured traits, the chitosan × NCC interaction was nonsignificant for CAT, TCC, and CAR, with significant variation observed only in the remaining parameters (Table S2). Supported by Bonferroni post hoc analysis, the application of 0.4% chitosan to unstressed plants significantly enhanced all physiological and morphological parameters relative to the mock control (Figure 4, green indicators). NCC application at 1000 and 2000 mg L^−1^ resulted in nonsignificant differences for FDS, LDS, EC, TCC, POX, SOD, and CAT compared to the mock. However, 1000 mg L^−1^ NCC significantly increased CAR and PRO (Figure 5, green indicators). The co‐application of NCC and chitosan to unstressed plants significantly enhanced FDS, LDS, TCC, EC, PRO, POX, CAR, and SOD relative to the mock (Tables 1, 2, 3, 4, “♦” symbol). CAT was significantly enhanced only under the 1000 mg L^−1^ NCC + chitosan treatment. These tissue‐based enhancements are illustrated in Figure 6 and Figure S4.

**FIGURE 6:**
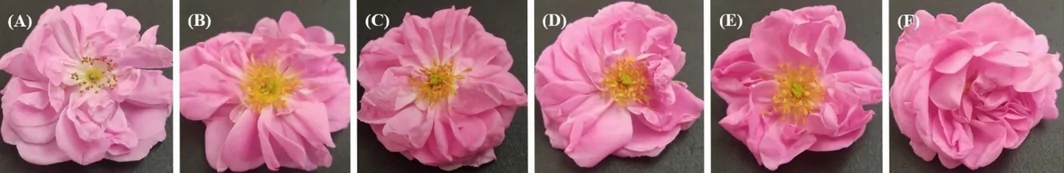
Comparative phenotypic plasticity of floral specimens of *Rosa × damascena* under foliar applications of 0.4% chitosan (Ch) or nano‐encapsulated copper formulations at two dosage levels of 1000 mg L^−1^ (NCC1) and 2000 mg L^−1^ (NCC2) concentrations. (A) Foliar applications of sterile distilled water; (B) Ch; (C) NCC1; (D) NCC2; (E) Ch + NCC1; (F) Ch + NCC2. A comparative phenotypic evaluation of leaves is presented in Figure S4. Photos are original works by the authors.

## 4 Discussion

The present study demonstrates that *R. × damascena* physiology and morphology are significantly modulated by the interactive effects of salinity, *B. cinerea* infection, and the application of chitosan and NCC. Rather than acting additively, these factors exhibited complex interactions, where the efficacy of mitigation strategies depended heavily on the prevailing abiotic and biotic stress background. Salinity stress imposed a significant physiological burden on *R. × damascena*, characterized by dose‐dependent increases in EC and disease severity (LDS and FDS), alongside reductions in photosynthetic pigments (TCC and CAR). These results align with established reports of salinity‐induced impairment in *Rosa* spp., where osmotic stress restricts water uptake, induces foliar desiccation, and disrupts nutrient allocation (Hura et al. 2017; Rehman et al. 2014; Oki and Lieth 2004). The observed decline in TCC and CAR under high salinity (100 mM) indicates a deterioration of photosynthetic capacity and photoprotection, consistent with chlorophyll degradation and disrupted thylakoid organization reported in other salt‐sensitive species (Bilal et al. 2020; Narayan et al. 2024).

Crucially, salinity did not act in isolation but significantly exacerbated *B. cinerea* severity. The combined stress treatment resulted in higher disease indices and EC than either stressor alone, suggesting a nonadditive coupling of abiotic and biotic stress. This supports the concept that abiotic stress modifies host–pathogen compatibility by weakening preformed barriers and diverting energy from inducible defenses (Tiwari et al. 2024). In this context, salinity may compromise membrane integrity, thereby facilitating fungal entry and colonization (Reis et al. 2016). However, it is important to note that the present study defines an ordinal response to coded salinity levels rather than a definitive salt‐tolerance threshold, as actual root‐zone EC and ion composition were not monitored. Consequently, the observed physiological thresholds and disease responses are specific to the coded salinity levels, the *B. cinerea* BS20 isolate, and the *R. × damascena* cultivar used in this study. A notable physiological finding was the decline in PRO and antioxidant enzyme activities at the highest salinity level. While PRO accumulation reflects both its role as an osmoprotectant and a stress biomarker, its decline under severe stress likely indicates a failure to sustain synthesis due to carbon limitation or tissue damage, rather than reduced stress perception (Ren et al. 2024). Thus, lower PRO at high stress conditions should be interpreted as a failure to sustain accumulation under prolonged or severe burden, rather than evidence of reduced stress perception.

The activity of antioxidant enzymes (SOD, POX, CAT) and EC served as key indicators of cellular redox status and membrane stability. Under individual *B. cinerea* infection, the significant reduction in CAT, POX, and SOD activities, coupled with elevated EC, suggests that the pathogen overwhelmed the host's enzymatic defense capacity, leading to membrane destabilization and oxidative injury. This correlates with reports of gray mold pathogenicity in ornamental roses, where infection leads to carbohydrate depletion and membrane damage (Ha et al. 2022). In contrast, treatments with chitosan and NCC generally restored or enhanced antioxidant activity. SOD, which converts superoxide radicals to H_2_O_2_, was significantly upregulated in treated plants, particularly under combined stress. However, the concurrent behavior of CAT and POX is critical for interpreting reactive oxygen species (ROS) detoxification. While SOD activity increased, CAT activity remained suppressed or unchanged in several combined stress treatments, indicating a potential bottleneck in H_2_O_2_ removal. Since CAT and peroxidases remove H_2_O_2_ in different cellular compartments (Baker et al. 2023), the lack of coordinated upregulation across all three enzymes suggests that the observed “protection” may be partial. Without direct measurements of ROS such as H_2_O_2_ or superoxide, and markers of lipid peroxidation like malondialdehyde, these enzyme activities represent potential antioxidant capacity rather than confirmed ROS detoxification flux (Zhang et al. 2024, 2025). Therefore, the interpretation of these biochemical markers remains indicative of stress status rather than definitive proof of metabolic recovery.

Both chitosan and NCC demonstrated protective effects, reducing disease severity and EC while maintaining pigment content. Chitosan likely functions through a dual mechanism: as a physical barrier or film‐forming agent that reduces water loss, and as a microbe‐associated molecular pattern analogue that primes host defense systems (Hassan et al. 2021). Perception of chitosan oligomers can initiate calcium fluxes, mitogen‐activated protein kinase cascades, and the expression of defense‐related genes, including those involved in phenylpropanoid metabolism and cell wall reinforcement (Rojas‐Pirela et al. 2024). This priming effect is supported by studies in other crops where chitosan improved membrane stability and upregulated antioxidant genes under stress (Bigham Soostani et al. 2025; Amooaghaie and Rajaie 2025). However, chitosan's efficacy is dose‐ and formulation‐dependent, and its antimicrobial properties may potentially inhibit beneficial microbiota if applied at excessive concentrations (Wulf et al. 2023). NCC application exhibited a nonlinear, hormetic response. The intermediate concentration (1000 mg L^−1^) generally provided the most favorable balance of reduced disease and enhanced physiological parameters, whereas the higher concentration (2000 mg L^−1^) showed diminishing returns or similar efficacy. This pattern is consistent with the dual role of copper as an essential micronutrient involved in redox enzymes and as a fungicidal agent that can cause phytotoxicity at high levels (Kaleem et al. 2024). Nano‐formulations may enhance bioavailability and adhesion, allowing for lower effective doses compared to conventional copper salts (Rawat et al. 2024; Adnan and Waleed 2025). Although tissue uptake was not directly measured, literature confirms that foliar‐applied nano‐chelated metals and chitosan penetrate cuticles and stomata in plants (Maluin and Hussein 2020; Xiong et al. 2017). However, the absence of tissue copper analysis prevents a definitive link between applied dose and internal copper status. Furthermore, without measuring soil or substrate copper accumulation, the long‐term environmental persistence and potential for copper toxicity in the rhizosphere remain unknown.

The significant interaction terms indicate that the efficacy of these amendments is context‐dependent. For instance, chitosan and NCC co‐application under moderate combined stress significantly reduced EC and enhanced SOD, but this effect was less pronounced or different under severe stress. This suggests that while these agents can mitigate stress, their capacity is limited by the severity of the underlying physiological damage. Furthermore, salinity‐induced stomatal closure likely restricted the foliar uptake of chitosan and NCC, particularly under high salinity conditions (Reis et al. 2016). This reduction in absorption efficiency may have contributed to the diminished mitigating effects observed under severe stress, highlighting the need for future studies to integrate gas exchange measurements with tissue accumulation analysis. The statistical interaction does not necessarily prove biological synergy in the strict sense (i.e., exceeding an additive null model), but it does confirm that the response to one input is modified by the presence of others. Potential synergistic mechanisms include chitosan stabilizing NCC particles to moderate copper release, while both agents contribute to membrane stabilization and defense priming (Al‐Khattaf et al. 2026; Farooq et al. 2022). However, caution is required when interpreting these interactions, as chitosan's antimicrobial nature or NCC could theoretically interfere with beneficial microbial consortia if they were present (Sunitha et al. 2026), although this was not a factor in the current sterile/fungal‐inoculation setup. Future studies should employ dose–response matrices and explicit additive models to rigorously test for synergy.

While the results demonstrate short‐term physiological benefits, claims of “sustainability” or “non‐toxicity” must be moderated. The seven‐day observation period is insufficient to rule out delayed phytotoxicity or cumulative copper accumulation in plant tissues, which is particularly relevant for *R. × damascena* given its use in essential oil and medicinal products (Wang 2024). Copper residues in harvested flowers could pose regulatory and safety concerns, and leaching into drainage water may impact soil health and nontarget organisms (López‐Prieto et al. 2023; Parada et al. 2024). Additionally, the repeated use of copper‐based nanomaterials may select for copper‐tolerant pathogen strains or disrupt beneficial soil microbiomes (Kamat and Kumari 2023). Therefore, these treatments should be viewed as components of an integrated management strategy rather than standalone sustainable fertilizers. Future research must prioritize factorial interaction analyses, direct pathogen measurements, comprehensive material characterization, and rigorous biosafety assessments. Multi‐season field validation is essential to evaluate efficacy, residual impacts, and economic viability before recommending these formulations for commercial rose production.

## Funding

This investigation was conducted exclusively utilizing institutional infrastructure and intramural resources accessible to the authors, without external financial support, grants, or in‐kind contributions from governmental agencies, private sector entities, or nonprofit organizations.

## Ethics Statement

The authors have nothing to report.

## Conflicts of Interest

The authors declare no conflicts of interest.

## Supporting information


**Figure S1:** In vitro antifungal activity of chitosan against *Botrytis cinerea*. Chitosan was dissolved in 1% (v/v) acetic acid and incorporated into Potato Dextrose Agar (PDA) to achieve final concentrations ranging from 0% (A), 0.04% (B), 0.08% (C), 0.12% (D), 0.16 (E), 0.2 (F), 0.24 (G), 0.28% (H), 0.32% (I), 0.36% (J), 0.4% (K), to 0.44% (L) with the pH maintained at approximately 5.6 to ensure polymer solubility. A 5 mm mycelial plug of *B. cinerea* (72 h old) was placed at the center of each plate to assess radial growth inhibition. The control treatment (A) consisted of PDA medium supplemented with 1% (v/v) acetic acid. Photographs taken by the authors.
**Figure S2:** Morphological responses of *Rosa × damascena* leaves to foliar treatments with 0.4% chitosan (Ch) and nano‐chelated copper at 1000 mg L^−1^ (NCC1) or 2000 mg L^−1^ (NCC2) under biotic (*Botrytis cinerea*; B.c) and abiotic (salinity: 50 mM [LS] or 100 mM [HS] NaCl/CaCl_2_) stressors. Panels illustrate specific treatment combinations: (A) B.c; (B) NCC1 + B.c; (C) NCC2 + B.c; (D) Ch + B.c; (E) Ch + NCC1 + B.c; (F) Ch + NCC2 + B.c; (G) LS; (H) NCC1 + LS; (I) NCC2 + LS; (J) Ch + LS; (K) Ch + NCC1 + LS; (L) Ch + NCC2 + LS; (M) HS; (N) NCC1 + HS; (O) NCC2 + HS; (P) Ch + HS; (Q) Ch + NCC1 + HS; (R) Ch + NCC2 + HS. Flower phenotype comparisons are detailed in Figure 1. All images presented are original photographs taken by the authors.
**Figure S3:** Visual documentation of leaf phenotype modulation in *Rosa × damascena* under concurrent salinity (50 mM [LS] or 100 mM [HS] NaCl/CaCl_2_) and *Botrytis cinerea* (B.c) stress, following exogenous application of 0.4% chitosan (Ch) and nano‐chelated copper at 1000 mg L^−1^ (NCC1) or 2000 mg L^−1^ (NCC2). Panels represent specific treatment combinations: (A) LS + B.c; (B) NCC1 + LS + B.c; (C) NCC2 + LS + B.c; (D) Ch + LS + B.c; (E) Ch + NCC1 + LS + B.c; (F) Ch + NCC2 + LS + B.c; (G) HS + B.c; (H) NCC1 + HS + B.c; (I) NCC2 + HS + B.c; (J) Ch + HS + B.c; (K) Ch + NCC1 + HS + B.c; (L) Ch + NCC2 + HS + B.c. Associated flower morphological responses are illustrated in Figure 3. Photos are original works by the authors.
**Figure S4:** Morphological characteristics of *Rosa × damascena* leaves following foliar supplementation with 0.4% chitosan (Ch) and nano‐chelated copper at 1000 mg L^−1^ (NCC1) or 2000 mg L^−1^ (NCC2) in the absence of stress. Panels illustrate specific treatment groups: (A) sterile distilled water control; (B) Ch; (C) NCC1; (D) NCC2; (E) Ch + NCC1; (F) Ch + NCC2. Corresponding leaf phenotypes are detailed in Figure S6. All images presented are original photographs taken by the authors.
**Table S1:** List of the 36 treatment combinations used in the different factorial experiment involving salinity, *Botrytis cinerea* infection, chitosan, and nano‐chelated copper applications (interaction indicated by “×”).
**Table S2:** Results of factorial ANOVA related to main and interaction effects of salinity, *Botrytis cinerea* infection, chitosan, and nano‐chelated copper on physiological and morphological traits of *Rosa × damascena*.

## Data Availability

The data that supports the findings of this study are available in the Supplementary Material of this article.
