# Supplementary material for: Modulation of Physiological Responses in Damask Rose to Combined Salinity and Botrytis cinerea Stress by Chitosan and Nano‐Chelated Copper
Source: Plant Environ Interact. 2026 Nov 10;7(6):e70243. doi: 10.1002/pei3.70243 (PMC13752395; doi:10.1002/pei3.70243)
Supplement: Supplementary file 1 [file PEI3-7-e70243-s001.docx]

**Figure S1.** *In vitro* antifungal activity of chitosan against *Botrytis cinerea*. Chitosan was dissolved in 1% (v/v) acetic acid and incorporated into Potato Dextrose Agar (PDA) to achieve final concentrations ranging from 0% **(A)**, 0.04% **(B)**, 0.08% **(C)**, 0.12% **(D)**, 0.16 **(E)**, 0.2 **(F)**, 0.24 **(G)**, 0.28% **(H)**, 0.32% **(I)**, 0.36% **(J)**, 0.4% **(K)**, to 0.44% **(L)** with the pH maintained at approximately 5.6 to ensure polymer solubility. A 5 mm mycelial plug of *B. cinerea* (72 h old) was placed at the center of each plate to assess radial growth inhibition. The control treatment (A) consisted of PDA medium supplemented with 1% (v/v) acetic acid. Photographs taken by the authors.

**Figure S2.** Morphological responses of *Rosa × damascena* leaves to foliar treatments with 0.4% chitosan (Ch) and nano-chelated copper at 1000 mg L⁻¹ (NCC1) or 2000 mg L⁻¹ (NCC2) under biotic (*Botrytis cinerea*; B.c) and abiotic (salinity: 50 mM [LS] or 100 mM [HS] NaCl/CaCl₂) stressors. Panels illustrate specific treatment combinations: **(A)** B.c; **(B)** NCC1 + B.c; **(C)** NCC2 + B.c; **(D)** Ch + B.c; **(E)** Ch + NCC1 + B.c; **(F)** Ch + NCC2 + B.c; **(G)** LS; **(H)** NCC1 + LS; **(I)** NCC2 + LS; **(J)** Ch + LS; **(K)** Ch + NCC1 + LS; **(L)** Ch + NCC2 + LS; **(M)** HS; **(N)** NCC1 + HS; **(O)** NCC2 + HS; **(P)** Ch + HS; **(Q)** Ch + NCC1 + HS; **(R)** Ch + NCC2 + HS. Flower phenotype comparisons are detailed in Fig. 1. All images presented are original photographs taken by the authors.

**Figure S3.** Visual documentation of leaf phenotype modulation in *Rosa × damascena* under concurrent salinity (50 mM [LS] or 100 mM [HS] NaCl/CaCl₂) and *Botrytis cinerea* (B.c) stress, following exogenous application of 0.4% chitosan (Ch) and nano-chelated copper at 1000 mg L⁻¹ (NCC1) or 2000 mg L⁻¹ (NCC2). Panels represent specific treatment combinations: **(A)** LS + B.c; **(B)** NCC1 + LS + B.c; **(C)** NCC2 + LS + B.c; **(D)** Ch + LS + B.c; **(E)** Ch + NCC1 + LS + B.c; **(F)** Ch + NCC2 + LS + B.c; **(G)** HS + B.c; **(H)** NCC1 + HS + B.c; **(I)** NCC2 + HS + B.c; **(J)** Ch + HS + B.c; **(K)** Ch + NCC1 + HS + B.c; **(L)** Ch + NCC2 + HS + B.c. Associated flower morphological responses are illustrated in Fig. 3. Photos are original works by the authors.

**Figure S4.** Morphological characteristics of *Rosa × damascena* leaves following foliar supplementation with 0.4% chitosan (Ch) and nano-chelated copper at 1000 mg L⁻¹ (NCC1) or 2000 mg L⁻¹ (NCC2) in the absence of stress. Panels illustrate specific treatment groups: **(A)** sterile distilled water control; **(B)** Ch; **(C)** NCC1; **(D)** NCC2; **(E)** Ch + NCC1; **(F)** Ch + NCC2. Corresponding leaf phenotypes are detailed in Figure S6. All images presented are original photographs taken by the authors.

**Table S1.** List of the 36 treatment combinations used in the different factorial experiment involving salinity, *Botrytis cinerea* infection, chitosan, and nano-chelated copper applications (interaction indicated by “**×**”)

| **Treatment** | **Factorial design** | | | | | | | | | | | | | | |
| --- | --- | --- | --- | --- | --- | --- | --- | --- | --- | --- | --- | --- | --- | --- | --- |
|  | Salinity | *Botrytis cinerea* | Chitosan | Nano-chelated copper | Salinity × *B. cinerea* | Salinity × Chitosan | Salinity × Nano-chelated copper | *B. cinerea* × Chitosan | *B. cinerea* × Nano-chelated copper | Chitosan × Nano-chelated copper | Salinity × *B. cinerea* × Chitosan | Salinity × *B. cinerea* × Nano-chelated copper | Salinity × Chitosan × Nano-chelated copper | *B. cinerea* × Chitosan × Nano-chelated copper | Salinity × *B. cinerea* × Chitosan × Nano-chelated copper |
| M | ● | ● | ● | ● | ● | ● | ● | ● | ● | ● | ● | ● | ● | ● | ● |
| LS | ● |  |  |  | ● | ● | ● |  |  |  | ● | ● | ● |  | ● |
| HS | ● |  |  |  | ● | ● | ● |  |  |  | ● | ● | ● |  | ● |
| B.c |  | ● |  |  | ● |  |  | ● | ● |  | ● | ● |  | ● | ● |
| LS + B.c |  |  |  |  | ● |  |  |  |  |  | ● | ● |  |  | ● |
| HS + B.c |  |  |  |  | ● |  |  |  |  |  | ● | ● |  |  | ● |
| Ch |  |  | ● |  |  | ● |  | ● |  | ● | ● |  | ● | ● | ● |
| LS + Ch |  |  |  |  |  | ● |  |  |  |  | ● |  | ● |  | ● |
| HS + Ch |  |  |  |  |  | ● |  |  |  |  | ● |  | ● |  | ● |
| B.c + Ch |  |  |  |  |  |  |  | ● |  |  | ● |  |  | ● | ● |
| LS + B.c + Ch |  |  |  |  |  |  |  |  |  |  | ● |  |  |  | ● |
| HS + B.c + Ch |  |  |  |  |  |  |  |  |  |  | ● |  |  |  | ● |
| NCC1 |  |  |  | ● |  |  | ● |  | ● | ● |  | ● | ● | ● | ● |
| LS + NCC1 |  |  |  |  |  |  | ● |  |  |  |  | ● | ● |  | ● |
| HS + NCC1 |  |  |  |  |  |  | ● |  |  |  |  | ● | ● |  | ● |
| B.c + NCC1 |  |  |  |  |  |  |  |  | ● |  |  | ● |  | ● | ● |
| LS + B.c + NCC1 |  |  |  |  |  |  |  |  |  |  |  | ● |  |  | ● |
| HS + B.c + NCC1 |  |  |  |  |  |  |  |  |  |  |  | ● |  |  | ● |
| NCC1 + Ch |  |  |  |  |  |  |  |  |  | ● |  |  | ● | ● | ● |
| LS + Ch + NCC1 |  |  |  |  |  |  |  |  |  |  |  |  | ● |  | ● |
| HS + Ch + NCC1 |  |  |  |  |  |  |  |  |  |  |  |  | ● |  | ● |
| B.c + Ch + NNC1 |  |  |  |  |  |  |  |  |  |  |  |  |  | ● | ● |
| LS + B.c + Ch + NCC1 |  |  |  |  |  |  |  |  |  |  |  |  |  |  | ● |
| HS + B.c + Ch + NCC1 |  |  |  |  |  |  |  |  |  |  |  |  |  |  | ● |
| NCC2 |  |  |  | ● |  |  | ● |  | ● | ● |  | ● | ● | ● | ● |
| LS + NCC2 |  |  |  |  |  |  | ● |  |  |  |  | ● | ● |  | ● |
| HS + NCC2 |  |  |  |  |  |  | ● |  |  |  |  | ● | ● |  | ● |
| B.c + NCC2 |  |  |  |  |  |  |  |  | ● |  |  | ● |  | ● | ● |
| LS + B.c + NCC2 |  |  |  |  |  |  |  |  |  |  |  | ● |  |  | ● |
| HS + B.c + NCC2 |  |  |  |  |  |  |  |  |  |  |  | ● |  |  | ● |
| NCC2 + Ch |  |  |  |  |  |  |  |  |  | ● |  |  | ● | ● | ● |
| LS + Ch + NCC2 |  |  |  |  |  |  |  |  |  |  |  |  | ● |  | ● |
| HS + Ch + NCC2 |  |  |  |  |  |  |  |  |  |  |  |  | ● |  | ● |
| B.c + Ch + NNC2 |  |  |  |  |  |  |  |  |  |  |  |  |  | ● | ● |
| LS + B.c + Ch + NCC2 |  |  |  |  |  |  |  |  |  |  |  |  |  |  | ● |
| HS + B.c + Ch + NCC2 |  |  |  |  |  |  |  |  |  |  |  |  |  |  | ● |

The experimental treatment comprised a mock control (M), *B. cinerea* infection (B.c), two salinity regimes consisting of 50 mM (LS) and 100 mM NaCl/CaCl₂ (HS), as well as foliar sprays of 0.4% w/v chitosan (Ch) and nano-chelated copper at 1000 mg L⁻¹ (NCC1) or 2000 mg L⁻¹ (NCC2). Combined treatments are denoted by the "+" symbol. The “●” symbol corresponds to the factorial arrangement.

**Table S2.** Results of factorial ANOVA related to main and interaction effects of salinity, *Botrytis cinerea* infection, chitosan, and nano-chelated copper on physiological and morphological traits of *Rosa × damascena*

| **Source** | | **Type III Sum of Squares** | **df** | **Mean Square** | **F** | **Sig.** |
| --- | --- | --- | --- | --- | --- | --- |
| Corrected Model | Peroxidase | .000^a^ | 35 | 9.329E-06 | 30.545 | 0.000 |
|  | Superoxide dismutase | 43934.811^b^ | 35 | 1255.280 | 31.648 | 0.000 |
|  | Catalase | 6.029^c^ | 35 | 0.172 | 9.051 | 0.000 |
|  | Total chlorophyll | 18.319^d^ | 35 | 0.523 | 26.134 | 0.000 |
|  | Carotenoid | 16.169^e^ | 35 | 0.462 | 19.266 | 0.000 |
|  | Proline | 5.333^f^ | 35 | 0.152 | 21.642 | 0.000 |
|  | Electrical conductivity | 39877.981^g^ | 35 | 1139.371 | 153.538 | 0.000 |
|  | Disease severity of leaf | 192.381^h^ | 35 | 5.497 | 83.426 | 0.000 |
|  | Disease severity of flower | 203.449^i^ | 35 | 5.813 | 94.512 | 0.000 |
| Intercept | Peroxidase | 0.002 | 1 | 0.002 | 6480.985 | 0.000 |
|  | Superoxide dismutase | 425254.240 | 1 | 425254.240 | 10721.437 | 0.000 |
|  | Catalase | 2.188 | 1 | 2.188 | 114.949 | 0.000 |
|  | Total chlorophyll | 308.865 | 1 | 308.865 | 15421.807 | 0.000 |
|  | Carotenoid | 328.863 | 1 | 328.863 | 13714.782 | 0.000 |
|  | Proline | 275.233 | 1 | 275.233 | 39091.541 | 0.000 |
|  | Electrical conductivity | 207336.962 | 1 | 207336.962 | 27939.995 | 0.000 |
|  | Disease severity of leaf | 589.494 | 1 | 589.494 | 8947.171 | 0.000 |
|  | Disease severity of flower | 518.505 | 1 | 518.505 | 8430.460 | 0.000 |
| Salinity | Peroxidase | 4.126E-05 | 2 | 2.063E-05 | 67.548 | 0.000 |
|  | Superoxide dismutase | 7411.413 | 2 | 3705.706 | 93.428 | 0.000 |
|  | Catalase | 1.980 | 2 | 0.990 | 52.031 | 0.000 |
|  | Total chlorophyll | 4.415 | 2 | 2.207 | 110.217 | 0.000 |
|  | Carotenoid | 4.469 | 2 | 2.235 | 93.193 | 0.000 |
|  | Proline | 1.298 | 2 | 0.649 | 92.168 | 0.000 |
|  | Electrical conductivity | 8118.932 | 2 | 4059.466 | 547.039 | 0.000 |
|  | Disease severity of leaf | 53.960 | 2 | 26.980 | 409.491 | 0.000 |
|  | Disease severity of flower | 47.665 | 2 | 23.832 | 387.496 | 0.000 |
| *Botrytis cinerea* | Peroxidase | 6.607E-05 | 1 | 6.607E-05 | 216.324 | 0.000 |
|  | Superoxide dismutase | 12355.506 | 1 | 12355.506 | 311.505 | 0.000 |
|  | Catalase | 1.439 | 1 | 1.439 | 75.586 | 0.000 |
|  | Total chlorophyll | 6.710 | 1 | 6.710 | 335.038 | 0.000 |
|  | Carotenoid | 4.490 | 1 | 4.490 | 187.234 | 0.000 |
|  | Proline | 1.655 | 1 | 1.655 | 235.083 | 0.000 |
|  | Electrical conductivity | 13895.583 | 1 | 13895.583 | 1872.520 | 0.000 |
|  | Disease severity of leaf | 75.401 | 1 | 75.401 | 1144.407 | 0.000 |
|  | Disease severity of flower | 87.768 | 1 | 87.768 | 1427.040 | 0.000 |
| Chitosan | Peroxidase | 6.698E-05 | 1 | 6.698E-05 | 219.305 | 0.000 |
|  | Superoxide dismutase | 8935.110 | 1 | 8935.110 | 225.270 | 0.000 |
|  | Catalase | 0.253 | 1 | 0.253 | 13.297 | 0.000 |
|  | Total chlorophyll | 3.315 | 1 | 3.315 | 165.495 | 0.000 |
|  | Carotenoid | 1.396 | 1 | 1.396 | 58.230 | 0.000 |
|  | Proline | 1.007 | 1 | 1.007 | 143.063 | 0.000 |
|  | Electrical conductivity | 7719.584 | 1 | 7719.584 | 1040.264 | 0.000 |
|  | Disease severity of leaf | 22.304 | 1 | 22.304 | 338.526 | 0.000 |
|  | Disease severity of flower | 26.641 | 1 | 26.641 | 433.164 | 0.000 |
| Nano chelated copper | Peroxidase | 7.808E-05 | 2 | 3.904E-05 | 127.829 | 0.000 |
|  | Superoxide dismutase | 8845.239 | 2 | 4422.619 | 111.502 | 0.000 |
|  | Catalase | 0.139 | 2 | 0.070 | 3.658 | 0.031 |
|  | Total chlorophyll | 2.345 | 2 | 1.173 | 58.550 | 0.000 |
|  | Carotenoid | 1.093 | 2 | 0.546 | 22.786 | 0.000 |
|  | Proline | 0.832 | 2 | 0.416 | 59.099 | 0.000 |
|  | Electrical conductivity | 6218.419 | 2 | 3109.210 | 418.986 | 0.000 |
|  | Disease severity of leaf | 20.399 | 2 | 10.199 | 154.802 | 0.000 |
|  | Disease severity of flower | 20.664 | 2 | 10.332 | 167.992 | 0.000 |
| Salinity × *B. cinerea* | Peroxidase | 6.046E-06 | 2 | 3.023E-06 | 9.898 | 0.000 |
|  | Superoxide dismutase | 234.844 | 2 | 117.422 | 2.960 | 0.058 |
|  | Catalase | 1.562 | 2 | 0.781 | 41.041 | 0.000 |
|  | Total chlorophyll | 0.573 | 2 | 0.287 | 14.315 | 0.000 |
|  | Carotenoid | 2.148 | 2 | 1.074 | 44.791 | 0.000 |
|  | Proline | 0.124 | 2 | 0.062 | 8.829 | 0.000 |
|  | Electrical conductivity | 61.191 | 2 | 30.595 | 4.123 | 0.020 |
|  | Disease severity of leaf | 6.004 | 2 | 3.002 | 45.566 | 0.000 |
|  | Disease severity of flower | 2.924 | 2 | 1.462 | 23.768 | 0.000 |
| Salinity × Chitosan | Peroxidase | 1.639E-06 | 2 | 8.193E-07 | 2.683 | 0.075 |
|  | Superoxide dismutase | 208.902 | 2 | 104.451 | 2.633 | 0.079 |
|  | Catalase | 0.151 | 2 | 0.076 | 3.968 | 0.023 |
|  | Total chlorophyll | 0.025 | 2 | 0.013 | 0.629 | 0.536 |
|  | Carotenoid | 0.266 | 2 | 0.133 | 5.548 | 0.006 |
|  | Proline | 0.003 | 2 | 0.001 | 0.199 | 0.820 |
|  | Electrical conductivity | 138.667 | 2 | 69.334 | 9.343 | 0.000 |
|  | Disease severity of leaf | 1.546 | 2 | 0.773 | 11.733 | 0.000 |
|  | Disease severity of flower | 0.592 | 2 | 0.296 | 4.815 | 0.011 |
| Salinity × Nano chelated copper | Peroxidase | 2.125E-06 | 4 | 5.312E-07 | 1.739 | 0.151 |
|  | Superoxide dismutase | 239.330 | 4 | 59.832 | 1.508 | 0.209 |
|  | Catalase | 0.050 | 4 | 0.012 | 0.652 | 0.627 |
|  | Total chlorophyll | 0.005 | 4 | 0.001 | 0.061 | 0.993 |
|  | Carotenoid | 0.403 | 4 | 0.101 | 4.198 | 0.004 |
|  | Proline | 0.030 | 4 | 0.007 | 1.061 | 0.382 |
|  | Electrical conductivity | 242.356 | 4 | 60.589 | 8.165 | 0.000 |
|  | Disease severity of leaf | 1.352 | 4 | 0.338 | 5.130 | 0.001 |
|  | Disease severity of flower | 1.477 | 4 | 0.369 | 6.003 | 0.000 |
| *B. cinerea* × Chitosan | Peroxidase | 1.885E-06 | 1 | 1.885E-06 | 6.174 | 0.015 |
|  | Superoxide dismutase | 405.848 | 1 | 405.848 | 10.232 | 0.002 |
|  | Catalase | 0.147 | 1 | 0.147 | 7.710 | 0.007 |
|  | Total chlorophyll | 0.137 | 1 | 0.137 | 6.817 | 0.011 |
|  | Carotenoid | 0.303 | 1 | 0.303 | 12.634 | 0.001 |
|  | Proline | 7.500E-05 | 1 | 7.500E-05 | 0.011 | 0.918 |
|  | Electrical conductivity | 103.449 | 1 | 103.449 | 13.940 | 0.000 |
|  | Disease severity of leaf | 0.015 | 1 | 0.015 | 0.230 | 0.633 |
|  | Disease severity of flower | 0.000 | 1 | 0.000 | 0.004 | 0.951 |
| *B. cinerea* × Nano chelated copper | Peroxidase | 1.541E-06 | 2 | 7.706E-07 | 2.523 | 0.087 |
|  | Superoxide dismutase | 550.986 | 2 | 275.493 | 6.946 | 0.002 |
|  | Catalase | 0.076 | 2 | 0.038 | 2.005 | 0.142 |
|  | Total chlorophyll | 0.048 | 2 | 0.024 | 1.210 | 0.304 |
|  | Carotenoid | 0.341 | 2 | 0.170 | 7.105 | 0.002 |
|  | Proline | 0.017 | 2 | 0.009 | 1.227 | 0.299 |
|  | Electrical conductivity | 27.820 | 2 | 13.910 | 1.874 | 0.161 |
|  | Disease severity of leaf | 0.065 | 2 | 0.033 | 0.494 | 0.612 |
|  | Disease severity of flower | 0.240 | 2 | 0.120 | 1.954 | 0.149 |
| Chitosan × Nano chelated copper | Peroxidase | 3.787E-05 | 2 | 1.893E-05 | 61.997 | 0.000 |
|  | Superoxide dismutase | 3249.751 | 2 | 1624.876 | 40.966 | 0.000 |
|  | Catalase | 0.006 | 2 | 0.003 | 0.161 | 0.852 |
|  | Total chlorophyll | 0.073 | 2 | 0.036 | 1.820 | 0.169 |
|  | Carotenoid | 0.130 | 2 | 0.065 | 2.715 | 0.073 |
|  | Proline | 0.088 | 2 | 0.044 | 6.268 | 0.003 |
|  | Electrical conductivity | 1137.899 | 2 | 568.949 | 76.670 | 0.000 |
|  | Disease severity of leaf | 2.411 | 2 | 1.206 | 18.298 | 0.000 |
|  | Disease severity of flower | 1.162 | 2 | 0.581 | 9.450 | 0.000 |
| Salinity × *B. cinerea* × Chitosan | Peroxidase | 2.007E-06 | 2 | 1.003E-06 | 3.286 | 0.043 |
|  | Superoxide dismutase | 123.045 | 2 | 61.522 | 1.551 | 0.219 |
|  | Catalase | 0.127 | 2 | 0.064 | 3.337 | 0.041 |
|  | Total chlorophyll | 0.058 | 2 | 0.029 | 1.449 | 0.242 |
|  | Carotenoid | 0.094 | 2 | 0.047 | 1.961 | 0.148 |
|  | Proline | 0.001 | 2 | 0.000 | 0.044 | 0.957 |
|  | Electrical conductivity | 507.359 | 2 | 253.680 | 34.185 | 0.000 |
|  | Disease severity of leaf | 3.465 | 2 | 1.733 | 26.298 | 0.000 |
|  | Disease severity of flower | 6.908 | 2 | 3.454 | 56.160 | 0.000 |
| Salinity × *B. cinerea* × Nano chelated copper | Peroxidase | 6.808E-06 | 4 | 1.702E-06 | 5.573 | 0.001 |
|  | Superoxide dismutase | 449.847 | 4 | 112.462 | 2.835 | 0.030 |
|  | Catalase | 0.036 | 4 | 0.009 | 0.471 | 0.757 |
|  | Total chlorophyll | 0.197 | 4 | 0.049 | 2.463 | 0.053 |
|  | Carotenoid | 0.344 | 4 | 0.086 | 3.588 | 0.010 |
|  | Proline | 0.063 | 4 | 0.016 | 2.224 | 0.075 |
|  | Electrical conductivity | 789.666 | 4 | 197.416 | 26.603 | 0.000 |
|  | Disease severity of leaf | 4.530 | 4 | 1.133 | 17.189 | 0.000 |
|  | Disease severity of flower | 6.399 | 4 | 1.600 | 26.012 | 0.000 |
| Salinity × Chitosan × Nano chelated copper | Peroxidase | 3.405E-06 | 4 | 8.513E-07 | 2.787 | 0.033 |
|  | Superoxide dismutase | 282.880 | 4 | 70.720 | 1.783 | 0.142 |
|  | Catalase | 0.028 | 4 | 0.007 | 0.370 | 0.829 |
|  | Total chlorophyll | 0.222 | 4 | 0.055 | 2.766 | 0.034 |
|  | Carotenoid | 0.264 | 4 | 0.066 | 2.752 | 0.034 |
|  | Proline | 0.098 | 4 | 0.025 | 3.494 | 0.012 |
|  | Electrical conductivity | 95.501 | 4 | 23.875 | 3.217 | 0.017 |
|  | Disease severity of leaf | 0.351 | 4 | 0.088 | 1.331 | 0.267 |
|  | Disease severity of flower | 0.870 | 4 | 0.217 | 3.535 | 0.011 |
| *B. cinerea* × Chitosan × Nano chelated copper | Peroxidase | 1.887E-06 | 2 | 9.436E-07 | 3.090 | 0.052 |
|  | Superoxide dismutase | 348.075 | 2 | 174.037 | 4.388 | 0.016 |
|  | Catalase | 0.005 | 2 | 0.003 | 0.140 | 0.870 |
|  | Total chlorophyll | 0.024 | 2 | 0.012 | 0.606 | 0.548 |
|  | Carotenoid | 0.187 | 2 | 0.094 | 3.903 | 0.025 |
|  | Proline | 0.014 | 2 | 0.007 | 1.014 | 0.368 |
|  | Electrical conductivity | 38.764 | 2 | 19.382 | 2.612 | 0.080 |
|  | Disease severity of leaf | 0.328 | 2 | 0.164 | 2.488 | 0.090 |
|  | Disease severity of flower | 0.088 | 2 | 0.044 | 0.719 | 0.491 |
| Salinity × *B. cinerea* × Chitosan × Nano chelated copper | Peroxidase | 8.910E-06 | 4 | 2.228E-06 | 7.294 | 0.000 |
|  | Superoxide dismutase | 294.035 | 4 | 73.509 | 1.853 | 0.128 |
|  | Catalase | 0.029 | 4 | 0.007 | 0.385 | 0.819 |
|  | Total chlorophyll | 0.172 | 4 | 0.043 | 2.142 | 0.084 |
|  | Carotenoid | 0.241 | 4 | 0.060 | 2.515 | 0.049 |
|  | Proline | 0.102 | 4 | 0.026 | 3.626 | 0.010 |
|  | Electrical conductivity | 782.790 | 4 | 195.697 | 26.371 | 0.000 |
|  | Disease severity of leaf | 0.251 | 4 | 0.063 | 0.951 | 0.440 |
|  | Disease severity of flower | 0.049 | 4 | 0.012 | 0.198 | 0.939 |
| Error | Peroxidase | 2.199E-05 | 72 | 3.054E-07 |  |  |
|  | Superoxide dismutase | 2855.802 | 72 | 39.664 |  |  |
|  | Catalase | 1.370 | 72 | 0.019 |  |  |
|  | Total chlorophyll | 1.442 | 72 | 0.020 |  |  |
|  | Carotenoid | 1.726 | 72 | 0.024 |  |  |
|  | Proline | 0.507 | 72 | 0.007 |  |  |
|  | Electrical conductivity | 534.297 | 72 | 7.421 |  |  |
|  | Disease severity of leaf | 4.744 | 72 | 0.066 |  |  |
|  | Disease severity of flower | 4.428 | 72 | 0.062 |  |  |
| Total | Peroxidase | 0.002 | 108 |  |  |  |
|  | Superoxide dismutase | 472044.854 | 108 |  |  |  |
|  | Catalase | 9.587 | 108 |  |  |  |
|  | Total chlorophyll | 328.625 | 108 |  |  |  |
|  | Carotenoid | 346.758 | 108 |  |  |  |
|  | Proline | 281.074 | 108 |  |  |  |
|  | Electrical conductivity | 247749.240 | 108 |  |  |  |
|  | Disease severity of leaf | 786.619 | 108 |  |  |  |
|  | Disease severity of flower | 726.381 | 108 |  |  |  |
| Corrected Total | Peroxidase | 0.000 | 107 |  |  |  |
|  | Superoxide dismutase | 46790.614 | 107 |  |  |  |
|  | Catalase | 7.399 | 107 |  |  |  |
|  | Total chlorophyll | 19.761 | 107 |  |  |  |
|  | Carotenoid | 17.896 | 107 |  |  |  |
|  | Proline | 5.840 | 107 |  |  |  |
|  | Electrical conductivity | 40412.278 | 107 |  |  |  |
|  | Disease severity of leaf | 197.125 | 107 |  |  |  |
|  | Disease severity of flower | 207.877 | 107 |  |  |  |
| a. R Squared = .937 (Adjusted R Squared = .906) | | | | | | |
| b. R Squared = .939 (Adjusted R Squared = .909) | | | | | | |
| c. R Squared = .815 (Adjusted R Squared = .725) | | | | | | |
| d. R Squared = .927 (Adjusted R Squared = .892) | | | | | | |
| e. R Squared = .904 (Adjusted R Squared = .857) | | | | | | |
| f. R Squared = .913 (Adjusted R Squared = .871) | | | | | | |
| g. R Squared = .987 (Adjusted R Squared = .980) | | | | | | |
| h. R Squared = .976 (Adjusted R Squared = .964) | | | | | | |
| i. R Squared = .979 (Adjusted R Squared = .968) | | | | | | |
